# Differential expression of genes identified by suppression subtractive hybridization in liver and adipose tissue of gerbils with diabetes

**DOI:** 10.1371/journal.pone.0191212

**Published:** 2018-02-02

**Authors:** Jingjing Gong, Xiaoyan Du, Zhenkun Li, Xiaohong Li, Meng Guo, Jing Lu, Ying Wang, Zhenwen Chen, Changlong Li

**Affiliations:** 1 School of Basic Medical Sciences, Capital Medical University, Beijing, China; 2 Department of Laboratory Animal, Capital Medical University, Beijing, China; Max Delbruck Centrum fur Molekulare Medizin Berlin Buch, GERMANY

## Abstract

**Objectives:**

We aimed at identifying genes related to hereditary type 2 diabetes expressed in the liver and the adipose tissue of spontaneous diabetic gerbils using suppression subtractive hybridization (SSH) screening.

**Methods:**

Two gerbil littermates, one with high and the other with normal blood glucose level, from our previously bred spontaneous diabetic gerbil strain were used in this study. To identify differentially expressed genes in the liver and the adipose tissue, mRNA from these tissues was extracted and SSH libraries were constructed for screening. After sequencing and BLAST analyzing, up or down-regulated genes possibly involved in metabolism and diabetes were selected, and their expression levels in diabetic gerbils and normal controls were analyzed using quantitative RT-PCR and Western blotting.

**Results:**

A total of 4 SSH libraries were prepared from the liver and the adipose tissue of gerbils. There are 95 up or down-regulated genes were identified to be involved in metabolism, oxidoreduction, RNA binding, cell proliferation, and differentiation or other function. Expression of 17 genes most possibly associated with diabetes was analyzed and seven genes (*Sardh*, *Slc39a7*, *Pfn1*, *Arg1*, *Cth*, *Sod1* and *P4hb*) in the liver and one gene (*Fabp4*) in the adipose tissue were identified that were significantly differentially expressed between diabetic gerbils and control animals.

**Conclusions:**

We identified eight genes associated with type 2 diabetes from the liver and the adipose tissue of gerbils via SSH screening. These findings provide further insights into the molecular mechanisms of diabetes and imply the value of our spontaneous diabetic gerbil strain as a diabetes model.

## Introduction

Diabetes mellitus (DM) is a severe human disease and is the fourth cause of mortality. Type 2 diabetes is the most common form of diabetes and ranges from insulin resistance, relative insulin deficiency to prevailing defective insulin secretion [1]. Genetic factors play major roles in the cause of type 2 diabetes [2]. Therefore, identifying and clarifying genetic factors underlying diabetes are of great importance.

It is essential to utilize animal models for investigation of genetic factors in diabetes research, especially spontaneous model. However, most existing DM animal models are inducible models or transgenic animal models. In chemical induction models chemical drugs (STZ, etc.) are administrated to injure the pancreatic β cells to inhibit insulin production. In the high-fat diet (HFD) induced type 2 diabetes model, vital organs are not damaged; however, it is more suitable to explore the role of nutrition factors in diabetes. Genetically modified animals, such as knock out of *IRS-1*, *IRS-2*, *GLUT-4*[3, 4], are limited to investigating pathogenic mechanisms because of dysfunction of one or two genes. Spontaneous models are produced without any conscious artificial manipulating. Hence, spontaneous diabetic models present hyperglycemia and insulin resistance and show more similar phenotypes and diabetes progression to human, therefore are more suitable for studying genetic factors underlying diabetes. However, spontaneous diabetes models are extremely precious and limited [5]. Fortunately, we established a spontaneous inbred diabetic Mongolian gerbil strain [6]. Mongolian gerbils (*Meriones unguiculatus*) are used as laboratory animals with a history of over 80 years [7, 8]. The possibility of using Mongolian gerbil as a diabetic model was first reported by Boquist who found that some gerbils in their colony had relatively higher fasting blood glucose and obesity [9].

On a standard laboratory diet, about 21.33% of animals in one subline of inbred gerbils showed an increased fasting blood glucose level and a decreased glucose tolerance, with diabetic damage in the liver and the adipose tissue. When inbreeding these gerbils to the 16^th^ generation, the fasting glucose levels, glucose tolerance, plasma insulin, and pathological analysis showed that a majority of these gerbils (60%) exhibited diabetic characteristics of FPG≥5.2 and PG2h ≥ 6.8 (mmol/l), as well as up-regulated insulin level [6]. It has been confirmed that this inbred strain of gerbil possesses the characteristics of hereditable type 2 diabetes. Thus, we think it would be an ideal animal model to investigate the genetic and molecular mechanism of diabetes. It is known that insulin resistance is the main characteristic of type 2 diabetes. We also observed insulin resistance, high homoeostasis model assessment for insulin resistance (HOMA-IR), and leptin resistance in our spontaneous diabetic gerbils [6]. However, pathological analysis showed that severe damages occurred in the liver not in the pancreas. The metabolic and alexipharmic activities of the liver and the endocrine activity of the adipose tissue are thought to be closely related to type 2 diabetes. The liver plays a unique role in controlling carbohydrate metabolism by regulating glucose concentration through hepatic glycogenesis, gluconeogenesis, and lipid synthesis [10]. It is reported that the prevalence of diabetes was higher in patients with liver diseases [11]. The type 2 diabetes is highly associated with the prevalence of NAFLD as well [12]. Therefore liver is considered an important organ in diabetes pathology. Meanwhile, the adipose tissue can produce a variety of hormones and cytokines including leptin and adiponectin which play a key role in the pathological processes of glucose metabolism, lipid metabolism in diabetes. Moreover, the adipose tissue is recognized as a primary site of insulin resistance and suppression of insulin signaling in adipocytes suffering from insulin resistance reduces glucose transport and metabolism [13]. Therefore, to identify genes that influence the development of diabetes in gerbils, we selected the liver and the adipose tissue as the subjects in our present study. We performed suppression subtractive hybridization (SSH) screening, a PCR-based method that amplifies differentially expressed cDNAs to isolate candidate genes expressed within distinct tissues [14], to identify potential genes associated with the characteristics of diabetes in gerbil.

## Materials and methods

### Ethics statement

All experiments and animal procedures were conducted in accordance with the Guidelines of the Capital Medical University Animal Experiments and the Experimental Animals Management Committee. The protocol was approved by the Animal Experiments and Experimental Animal Welfare Committee of Capital Medical University (Permit Number: AEEI-2017-032).

### Animal material

The liver and the adipose tissue from two inbred gerbils of the same litter (age 10 months) were isolated to build SSH libraries, and the same kinds of tissues from 10 other animals (aged 8–10 months) were used to verify the selected genes. We grouped gerbils into two categories: (1) gerbils with diabetes, exhibiting significantly higher blood glucose levels (FG 10.8 mmol/L) and some pathophysiological lesions, and (2) gerbils as controls with normal blood glucose level (FG 3.4 mmol/L) and no pathophysiological lesions. Of the 10 gerbils, half (five) suffered from diabetes, as determined by plasma glucose and blood insulin levels and histopathological analysis, and the remaining half (five) were controls with normal blood glucose and insulin levels without histopathological lesions. All animals were euthanized with an overdose of pentobarbital. The left and right lateral lobes of the liver and adipose from both sides of the groin were removed and stored in liquid nitrogen until use.

### Construction of SSH libraries

Four subtracted libraries were constructed (comprising two libraries each for pair of liver and adipose tissues from normal and diabetic gerbils) using SSH kit. Briefly, total RNA was extracted from frozen liver and adipose tissues using Trizol^®^ Reagent (Invitrogen, USA), and concentration and purity of RNA were measured using a Nanodrop 2000c (Thermo Scientific, USA). Total mRNA was purified using a Purification of poly (A) RNA kit (Macherey-Nagel, Germany). The mRNA samples were stored at −80°C prior to reverse transcription. cDNA synthesis, digestion with *Rsa*I, two hybridizations, and PCR amplification were performed using a PCR-Select cDNA Subtraction Kit (Clontech, USA) according to the manufacturer’s instructions. Subtractions were performed using a 5-fold excess amount of driver cDNA to tester cDNA. To obtain forward and reverse subtraction libraries from the two kinds of tissues, the resulting subtraction PCR products were purified and ligated into pMD19-T vector (TaKaRa, Japan), transformed into DH5α competent cells (ExCell, China), incubated at 37°C with shaking overnight, and plated onto agar plates containing ampicillin (Solarbio, China), X-gal (Takara, Japan), and isopropyl-β-d-thiogalactoside (IPTG; Takara, Japan). Positive clones of the four SSH libraries were sequenced using Sanger sequencing by Tianyi Huiyuan, China, then the BLAST (Basic Local Alignment Search Tool) analysis were performed.

### Gene expression in gerbils with diabetes analyzed using qPCR

After cDNA sequencing and BLAST analysis, all sequences got to obtained from the SSH libraries were classified and 17 genes most closely related to diabetes and metabolism disorders genes were selected. Their expression was analyzed in the diabetic and control gerbils using qPCR first. Livers and adipose tissues from 10 adult animals were used. Gene names and primers sequences (designed using Primer Premier5) are shown in Table 1.

**Table 1:** Gene names, primer sequences, annealing temperatures, and lengths of PCR products for qPCR analysis of 17 genes and two housekeeping genes (as referencing controls) related to diabetes and metabolism identified from SSH libraries.

Gene	Primer	Sequence	Annealing temperature (°C)	Product length (bp)
*β-actin*	Forward Primer	AGAGGGAAATCGTGCGTGAC	58	138
	Reverse Primer	CAATAGTGATGACCTGGCCGT		
*Gapdh*	Forward Primer	GCCATCAATGACCCC	58	103
	Reverse Primer	TCCCGTTCTCAGCCT		
*Sardh*	Forward Primer	AGCGACCTGACTGTTAGCC	58	142
	Reverse Primer	CCTGTAGCACCGTGTTTATG		
*Arg 1*	Forward Primer	ACCCAGTCTTCGGAACCTA	58	142
	Reverse Primer	GCCTGCCACCCGTAGTT		
*Slc39a7*	Forward Primer	CCTGGGTGATGCGTTCC	58	147
	Reverse Primer	GCGACAATCCCACTGAGAAC		
*P4hb*	Forward Primer	AAACTGGGCGAGACATAC	58	113
	Reverse Primer	AAGAACTTCAGCGTGGG		
*Pfn1*	Forward Primer	ATGGGCTGACACTTGGG	58	178
	Reverse Primer	CACCGTGGACACCTTCTTT		
*Fgb*	Forward Primer	TTTGGCAGGAAATGGG	58	126
	Reverse Primer	GGTGAGCTGGCTAATCTTAT		
*Cyp2e1*	Forward Primer	TATCCTTAGGGTCAACCA	58	131
	Reverse Primer	CTATTTCAAGCCATTTTCT		
*Sod1*	Forward Primer	CGAGCAGAAGGCAAGC	58	196
	Reverse Primer	GCCCAGGTCTCCAACA		
*Cth*	Forward Primer	GCATTTCGCCACTCAG	58	203
	Reverse Primer	TGTGCTTTGCCCCAT		
*Hpx*	Forward Primer	CCTTGAGGTGGCTTGA	58	158
	Reverse Primer	AGTTCCATTTCTGGGTCT		
*Tfr*	Forward Primer	AAAGATGGCGGTGGG	58	115
	Reverse Primer	GCTTGCGGGTATTGTC		
*Arfrp1*	Forward Primer	AAGAGGCATTTGAGAAGG	58	182
	Reverse Primer	TGTGAGGGCAGAGCAG		
*Apoa2*	Forward Primer	AAGGGCTCCGAGATTC	58	84
	Reverse Primer	TCCAGCTTGCTTGACC		
*Fabp4*	Forward Primer	TGGGAGTGGGCTTTG	63	199
	Reverse Primer	CCGCCATCTAGGGTTA		
*Nr1h2*	Forward Primer	ACCAGCCCAAAGTCACGC	58	126
	Reverse Primer	TTGGCAAAGTCCACAATCTCC		
*Ass1*	Forward Primer	TGCCAAAGCACCCA	63	160
	Reverse Primer	AATGCGACCCACTCC		

Total RNA was isolated from liver and adipose tissues using TRIzol reagent (Invitrogen, USA), and total RNA was dissolved in RNase-free ddH_2_O (Tiangen, China). First strand cDNA was synthesized from total RNA using a Fast Quant RT Kit (Tiangen, China). Quantitative real-time PCR was performed using SuperRealPreMix Plus (SYBR Green; Tiangen, China). We used qPCR with CFX96 manager (Bio-Rad, USA) and the following program: pre-denaturation at 95°C for 15 min, 40 cycles of denaturation at 95°C for 10 s, annealing and extension for 35 s, and detection of fluorescence intensity from 60°C to 95°C, by increasing the temperature at a rate of 0.5°C per second, using a melting curve analysis. The total volume of each qPCR sample was 20 μL. The relative levels of target gene expression were normalized to β-actin. Gene expression was calculated as 2^−ΔΔCt^.

### Western blotting analysis

After qPCR screening, nine potential diabetes-related proteins (seven from the liver and two from the adipose SSH library) were analyzed further using Western blotting. Because antibodies for these proteins in gerbils are not available, we assessed whether sequences from rat or mouse proteins were close to gerbil orthologues based on sequence distances and molecular phylogenetic analysis, and used this information to guide which antibodies would be suitable for use in Western blotting. The proteins were extracted using the Tissue Protein Extraction Kit (CWBIO, China) and quantified using BCA-Reagents (CWBIO, China). Protein lysates were subjected to sodium dodecyl sulfate-polyacrylamide gel electrophoresis (SDS-PAGE) at 160 V (Bio-Rad) on a 12% gel (CWBIO, China) for 50 min, transferred to a 0.22-μm nitrocellulose filter membrane (Solarbio, China) at 15 V for 20 min. The membrane was washed in TBS-Tween 20 for 5 min at 25°C then incubated in blocking buffer for 1 h at 25°C and washed in TBS-Tween 20 for three more times. The membrane was incubated overnight with primary antibodies at 4°Cwith gentle shaking and was then washed three times followed by incubation for 1 h at 25°C with the secondary antibody (Anti-rabbit IgG, Cell Signaling Technologies, USA). Finally, the membrane was visualized using chemiluminescence (ECL) immunoblotting detection reagents (Thermo Fisher Scientific, USA). Primary antibodies were: ARG1 and PFN1 (Cell Signaling Technologies, USA), 1:1000 dilution; FABP4, P4HB, SOD1, and CTH (Abcam, UK), 1:1000 dilution; SARDH and SLC39A7 (NOVUS, USA), 1:1000 dilution. Anti-rabbit IgG was diluted to 1:5000. Results were normalized to housekeeping genes GAPDH and β-actin after gray scanning.

### Statistical analysis

Statistical analysis was performed for all data using SPSS 16.0 (SPSS Inc., USA). Comparisons between different groups were calculated using Student’s t-test. Significance was assumed at *p* < 0.05. Error bars represent standard deviations.

## Results

### Construction of SSH libraries

We established four SSH libraries from the liver and the adipose tissue of two gerbils from the spontaneous diabetic gerbil strain, comprising one animal with high blood glucose level (diabetic gerbil) and its littermate with normal blood glucose as the control. All four libraries were constructed using the diabetic gerbil as the tester and the control as the driver; conversely, we also used the samples from diabetic gerbil as the driver and the control as the tester. The 4 SSH libraries contained nearly 2000 clones, half for the liver and another half for the adipose. These clones were sequenced until 20% of the obtained sequences were duplicates of previously sequenced genes. There are 59 genes up-regulated and 65 genes down-regulated in liver, and 52 genes up-regulated and 47 genes down-regulated in adipose. Thus, a total of 223 sequences from the liver and the adipose SSH libraries were selected for the following sequence BLAST analysis.

### Selection of diabetes-related genes in gerbil SSH libraries

We obtained 223 cDNA sequences in the range of 80–700 bp belonging to four libraries (up or down-regulated genes in the liver and the adipose). All the 223 sequences were analyzed using BLAST and we found 95 cDNA sequences of the 223 sequences belonging to named or known genes in GenBank, or described in previous studies, or had functional description in gerbil, human, rat or mouse. Of the 95 cDNA sequences, 78 derived from the liver SSH library and 17 from the adipose SSH library, respectively. The BLAST analysis identified 62 genes (65%, 62/95) as Expressed Sequence Tags (ESTs), and information about their function was available in related journal or from MGI (http://www.informatics.jax.org/) (S1 Table). These gerbil EST sequences were submitted to GenBank, and the dbEST ID and GenBank accession numbers (Accn) are shown in S1 Table.

**Fig 1:**
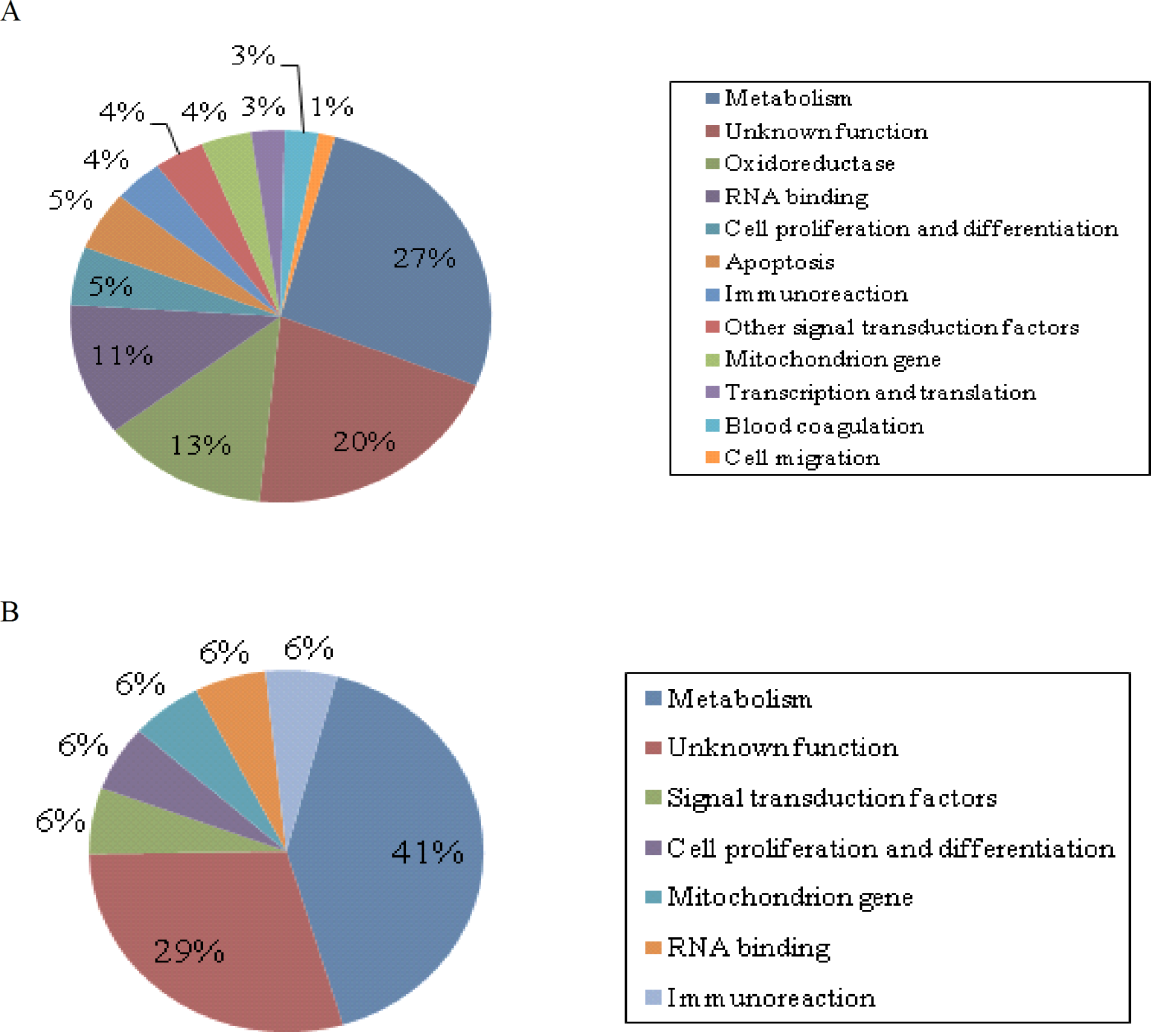
Classification of genes from suppression subtractive hybridization (SSH) libraries. (A) Putative functional classification of 78 genes from the liver SSH library for which identity was could be inferred. Information on function from previous reports or from the Mouse Genome Informatics database. These genes were distributed into 11 groups and were non-annotated based on their functions. (B) Seventeen known genes from the adipose SSH library were divided into seven groups according to their function. Methods were the same as in A.

Based on sequence similarities in NCBI databases, the 78 genes from the liver were subdivided into 12 functional categories (Fig 1A). Genes related to metabolism formed the largest category of differentially expressed genes (27%). Approximately 20% of the genes were novel genes in diabetic gerbil without clear functions. Oxidoreductase genes formed the third major category, accounting for 13% of all differentially expressed genes. RNA binding-related genes occupied an important category, making up to 11% (9/78) of the total genes. In contrast, genes belonging to the categories “cell proliferation and differentiation” and “apoptosis” accounted for a total of 5% and were slightly higher in number to the genes belonging to “immunoreaction,” “other signal transduction factors,” and “mitochondrion genes,” which comprised 4% of the total identified genes. Three percent of the total genes were related to transcription, translation, and blood coagulation. Finally, only 1% of all the identified genes were related to cell migration. The number of genes (17) identified from the adipose tissue was less than that from the liver (Fig 1B). However, metabolism-related genes still formed the largest category, comprising 41% of the total identified genes. About 29% of the total genes were novel genes, and thus, little information was available about these genes in the database. The remaining categories, cell proliferation and differentiation, signal transduction factors, mitochondrion genes, RNA binding, and immunoreactions-related genes, accounted for 6% of total genes. The largest category of the differentially expressed genes in both liver and adipose indicated that the main characteristics of this spontaneous diabetic gerbil are metabolic dysfunction and it would be a good model to explore the systemic metabolic disease.

### Evaluation of differential gene expression using quantitative real-time PCR

**Fig 2:**
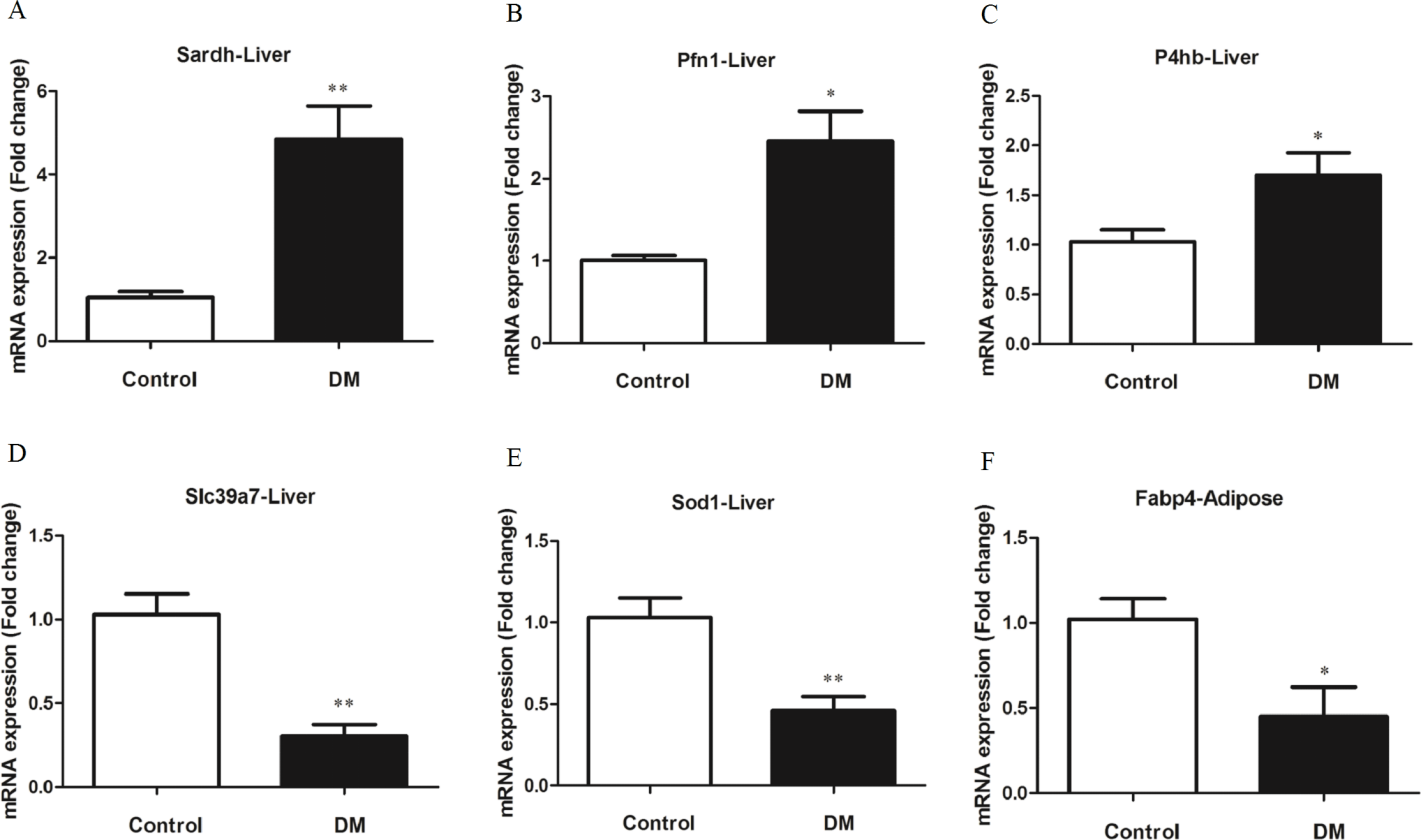
mRNA expression levels of six candidate genes selected from two SSH libraries from the liver and adipose tissue of gerbils. (A-E) Relative mRNA expression levels of five genes (*Sardh*, *Pfn1*, *P4hb*, *Slc39a7* and *Sod1*) between control and diabetic gerbils in the liver. (F) Relative mRNA expression levels of *Fabp4* between control and diabetic gerbils in adipose tissue. *p < 0.05, **p < 0.01.

To verify differential expression of the genes associated with diabetes, we selected 13 genes and 4 genes most closely relative to diabetes from the liver SSH library and the adipose SSH library, respectively (Table 2). The expression level of these candidate genes was examined in their corresponding tissue. We observed that the mRNA expression level of five genes (31.25%, 5/16) from the liver and one gene from the adipose tissue were significantly different between the diabetes and normal groups (Fig 2). The genes with the higher expression levels were identified from the tester in the corresponding SSH library, meaning that the expression was consistent with the original SSH library.

**Table 2:** Summary of 17 candidate genes and annotated functions.

Gene symbol	Name	Gene function	References
*Sardh*	Sarcosine dehydrogenase	Oxidoreduction	PMID:2417560
*Arg1*	Arginase 1	Hydrolyzing l-arginine in urea cycle	PMID:17645639
*Slc39a7*	Solute carrier family 39 member 7	Zinc transporter	PMID:24265765
*P4hb*	Prolyl 4-hydroxylase subunit beta	Endoplasmic reticulum molecular chaperone	PMID:8789253
*Pfn1*	Profiling 1	Binding protein	PMID:15939738
*Fgb*	Fibrinogen beta chain	Blood coagulation	PMID:10467729
*Cyp2e1*	Cytochrome P450 family 2 subfamily E member 1	Polar molecules metabolism	PMID:12537967
*Sod1*	Superoxide dismutase 1	Oxidoreduction	PMID:21603028
*Cth*	Cystathionine gamma-lyase	H_2_S metabolism	PMID:19019829
*Hpx*	Hemopexin	Binding heme	PMID:12042069
*Trf*	Transferring	Iron transport metabolism	PMID:18473900
*Arfrp1*	ADP ribosylation factor related protein 1	Intracellular protein traffic	PMID:17127620
*Apoa2*	Apolipoprotein A-II	Lipid metabolism	PMID:11714842
*Fabp4*	Fatty acid binding protein 4	Lipid metabolism	PMID:16054052
*Nr1h2*	Nuclear receptor subfamily 1 group H member 2	Lipid and carbohydrate metabolism	PMID:15831500
*Ass1*	Arginino succinate synthase 1	Arginine biosynthetic	PMID:3513483

Of the genes from the liver, the expression levels of *Sardh*, *Pfn1*, and *P4hb* (Fig 2A–2C) were significantly elevated in the group with diabetes. In contrast, the relative expression levels of *Slc39a7* and *Sod1* (Fig 2D and 2E) were significantly lower in the diabetic group than those in the control group. The relative expression levels of *Arg1*and *Cth* (S1A and S1B Fig) were suggestive of slightly lower expression in the diabetic samples, but we note this difference is not significant with current data. Of the four candidate genes in the adipose, the expression of *Fabp4* (Fig 2F) was decreased significantly in the group with diabetes. The mRNA expression levels of other genes identified in the liver and adipose tissue were not altered between the diabetes and control groups. Based on the results from both the liver and the adipose SSH libraries, we concluded that six genes were very possibly involved in diabetes in gerbils.

### Validation of candidate genes after qRT-PCR screening by Western blotting

After screening the expression of the genes associated with diabetes by qRT-PCR, we further examined whether the differences between the diabetes and control groups were also observed at the protein expression level in the liver and the adipose tissue. We selected 9 genes of which the mRNA expression tendencies were consistent with the original SSH library. First, we searched for the sequences of these genes in our rough *de novo* gerbil genomic sequencing data (data not published). We analyzed the open reading frame (ORF) sequences and compared them with the ORF from *Mus musculus*, *Rattus norvegicus*, *Sus scrofa*, and *Homo sapiens*. As expected, the ORF of most of these genes in gerbils was highly consistent with sequences from the other species, particularly mouse and rat. We performed Western blotting using mouse or rat antibodies to analyze protein expression in gerbil livers and adipose tissues.

**Fig 3:**
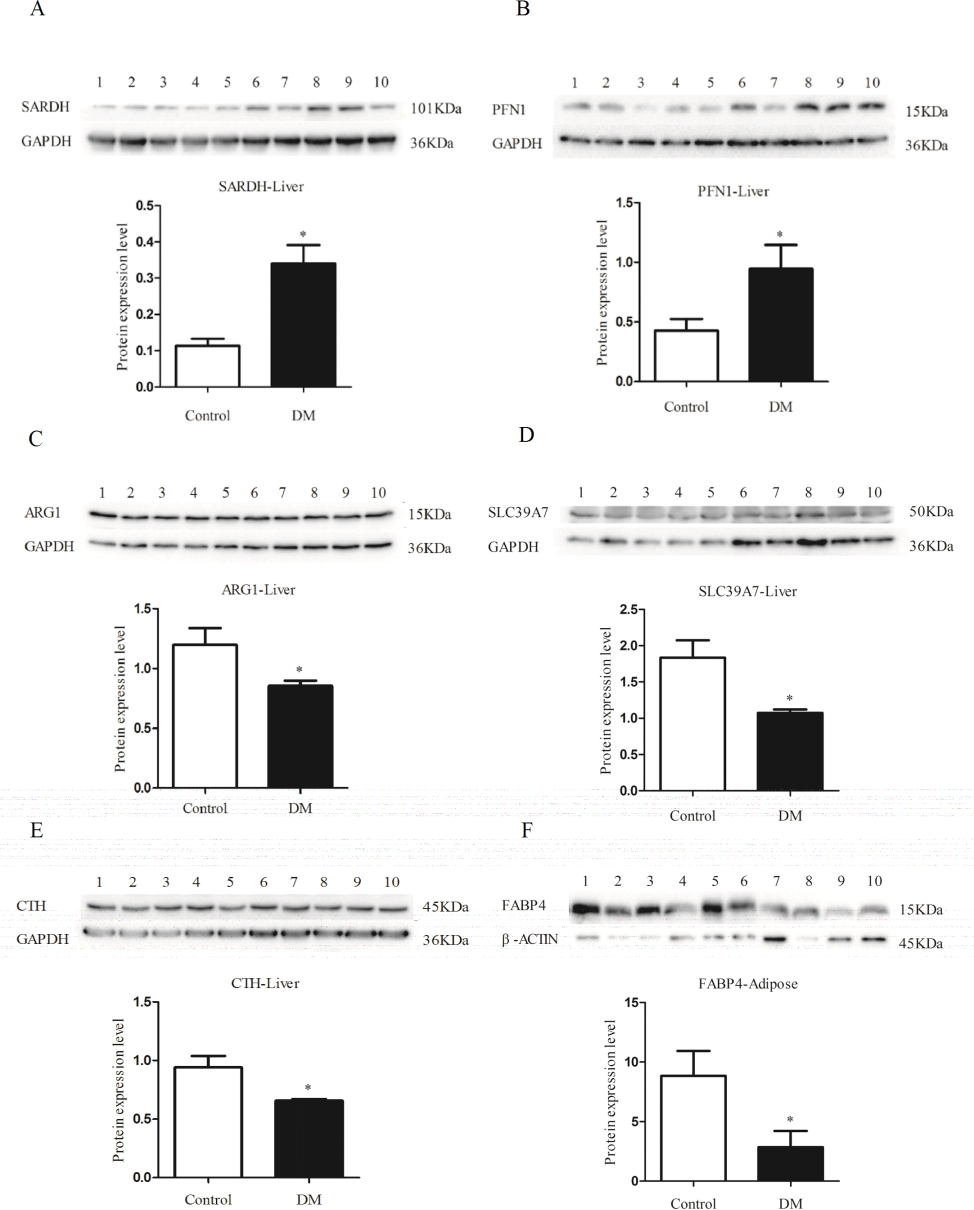
Expression levels of candidate proteins in liver and adipose tissue analyzed by Western blotting. (A-E) Protein expression levels of SARDH(A), PFN1(B), ARG1(C), SLC39A7(D), and CTH(E) in the liver of animals in the diabetes and control groups, as revealed by Western blot. (F) Protein levels of FABP4 in adipose tissues of gerbils with diabetes compared to controls. Control group is 1–5 and diabetic group is 6–10. Below the gray scan band, semi-quantitation of the data and corresponding statistics was determined using the Image J software. *p < 0.05.

We selected GAPDH as the internal control to standardize expression levels for genes in liver. Consistent with the qRT-PCR results for the liver, *Sardh* and *Pfn1* (Fig 3A and 3B) showed a higher expression level in gerbils with diabetes. Otherwise, *Arg1*, *Slc39a7*, and *Cth* (Fig 3C–3E) exhibited lower expression compared to the control. Despite the fact that significant differences in *Sod1 and P4hb* expression in the liver were observed in qRT-PCR, protein levels were similar between the diabetic and the control groups (S2 Fig). For the adipose tissue, we measured the expression level of *Fabp4* using *β-actin* as the internal control. The results (Fig 3F) demonstrated that *Fabp4* expression was significantly lower in the group with diabetes than that in controls. These results were consistent with those obtained from qRT-PCR and further confirmed that the six genes (*Sardh*, *Pfn1*, *Arg1*, *Slc39A7*, *Cth* and *Fabp4*) are associated with diabetes in gerbils.

## Discussion

We selected one pair of inbred gerbil littermates, one with high blood glucose level and diabetic features, including hyperglycemia, muscle degeneration necrosis, hepatic steatosis, fatty degeneration, and β-cell degeneration and the other one with normal characteristics[6]. SSH is a PCR-based method that can be used to identify differentially expressed genes in any two closely related samples, specimens, or species[15]. Therefore, the usage of littermates from the inbred diabetic gerbil strain approximately minimized genetic diversity and allowed us to focus more on the differential molecular characteristics of diabetes. Thus, the methods and the tissues used in the present study provided the possibility to identifying genes that were differentially expressed in diabetes or were associated with diabetes. We found that 95 genes of the 223 sequences from the four libraries have previously been described. More than half of the sequences were unreported, of which some may be novel diabetes-related genes. In addition, regardless of whether the genes were from the liver or adipose, the largest category of different expression genes belonged to metabolism and diabetes-related genes, confirming again that our diabetic gerbil is a metabolism dysfunction animal model.

Multiple genes have been reported to be involved in type 2 DM [16]. The candidate genes that we identified by SSH in the liver and the adipose tissues in the present study may also play important roles in the pathogenesis of diabetes. From the SSH libraries, a total of 17 genes were identified and verified using qPCR and Western blotting, and the results suggested that six genes (*Sardh*, *Slc39a7*, *Arg1*, *Pfn1*, *Cth*, *Fabp4*) may be associated with metabolism and diabetes in gerbils, with a further two genes having support from qPCR but not Western blotting analysis (Sod1, P4hb).

The gene *Sardh*, which showed a higher expression level in the gerbils with diabetes, is a flavoprotein that converts sarcosine back to glycine and catalyzes the conversion of dimethylglycine dehydrogenase (DMGDH) to sarcosine [17]. Although no reports have demonstrated that *Sardh* is directly related to diabetes, single nucleotide polymorphisms (SNPs) in *Sardh* associated with type 2 diabetes had been identified [18]. Another gene *Pfn1*, the expression of which increased in diabetic gerbils, is recognized as a binding protein involved in a variety of cellular activities [19]. Munkyong et al [20]demonstrated that a high-fat diet (HFD) upregulates *Pfn1* expression in white adipose tissue (WAT). Caglayan et al [21] reported that *Pfn1* expression is significantly enhanced in human atherosclerotic plaques compared with normal vessel walls. Therefore, our finding that the expression levels of *Sardh* and *Pfn1* were significantly higher in the livers of animals in gerbils with diabetes than those in the control animals indicates that these genes are associated with diabetes, particularly at the expression level. *Slc39a7* is a key zinc transporter that participates in the mechanisms of physiological and cellular zinc homeostasis and is abundantly expressed in human and mouse tissues [22]. When *Slc39a7* expression is reduced in skeletal muscles, several genes involved in glucose metabolism such as glycolysis and glycogen synthesis are altered [23]. *Slc39a7* was identified in our study, and significantly lower mRNA and protein levels in diabetic livers were observed, implying that *Slc39a7* plays a key role in the mechanism of diabetes in gerbils. We are currently further exploring the functions of these three new genes in diabetic gerbils by knocking them out using the clustered regularly interspaced short palindromic repeats (CRISPR)–CRISPR-associated protein-9 nuclease (Cas9) editing system.

One gene was identified in the adipose tissue of gerbils with diabetes: *Fabp4*, also known as adipocyte FABP (A-FABP) or aP2, which is abundantly expressed in adipocytes and may be a mediator of systemic insulin sensitivity and lipid and glucose metabolism [24]. *Fabp4* expression level was lower in animals with diabetes, which indicates that the gene is involved in the metabolism of diabetes in adipose tissues in gerbils.

We also confirmed other genes involved in diabetes, including *Cth*, *Arg1*, and *Sod1*. *Cth* encodes one of the major enzymes that catalyze hydrogen sulfide (H_2_S) in mammalian tissues, particularly in the liver [25]. Previous studies have shown that livers from STZ-treated T1D rats exhibit significantly decreased *Cth* mRNA, protein expression, enzymatic activity, and lower H_2_S formation compared to healthy controls [26]. It has also been reported that *Cth* expression was markedly decreased in the diabetic kidney with advanced lesions [27]. Lower expression levels of *Cth* in the diabetic liver observed in our study again confirmed the function of this gene in diabetes, similar to that reported for T2D rats [27]. *Arg1* is located in the cytoplasm and is expressed most abundantly in the liver of mammals. A previous study has reported that the expression of *Arg1* is up-regulated in the coronary arterioles of patients with DM [28]. However, *Arg1* expression was down-regulated in our model of diabetes, which was contradictory to the previous report [28]. A possible explanation for this could be the different animal models used in each study [29]. *Sod1* is an important component of apoptotic signaling and oxidative stress. In our study, *Sod1* expression was lower in gerbils with diabetes. Meanwhile, the effects of *Sod1* deficiency, such as oxidative damage impairing glucose tolerance and insulin sensitivity, have been reported [30]. P4HB, also known as protein disulfide isomerase, is a multifunctional protein and is a molecular chaperone that catalyzes the formation and rearrangement of disulfide bonds that help in ameliorating misfolded proteins in response to ER stress [31]. *P4hb* was upregulated in the diabetes group in our study. *Sod1* and *P4hb* showed significant differences in mRNA expression between the livers of gerbils with diabetes and control animals, implying that these genes play an important role in the mechanism of diabetes in gerbils.

In conclusion, we screened 95 genes that may be associated with diabetes in inbred gerbils using the SSH method. We also identified seven genes (*Sardh*, *Slc39a7*, *Pfn1*, *Arg1*, *Cth*, *Sod1 and P4hb*) in the liver and one gene (*Fabp4*) in the adipose tissue that may be associated with type 2 diabetes in gerbils. These newly identified sequences and genes may facilitate the development of further studies on diabetes and on the application of a gerbil model of diabetes.

## Supporting information

S1 Fig Two genes with no significant difference in mRNA expression levels between diabetes and control gerbils. (TIF)

S2 Fig Two genes with no significant difference in protein expression levels between diabetes and control gerbils. (TIF)

S1 Table User Id, dbEST Id, and GenBank Accn of ESTs from SSH libraries. (DOCX)

S1 File Un-altered western blot images of Fig 2, including the purpose bands and internal control bands of six genes. (ZIP)

S2 File Un-altered western blot images of S2 Fig, including the purpose bands and internal control bands of two genes. (ZIP)
