# Supplementary figures and images for: Differential expression of genes identified by suppression subtractive hybridization in liver and adipose tissue of gerbils with diabetes
Source: PLoS One. 2018 Feb 2;13(2):e0191212. doi: 10.1371/journal.pone.0191212 (PMC5796689; doi:10.1371/journal.pone.0191212)

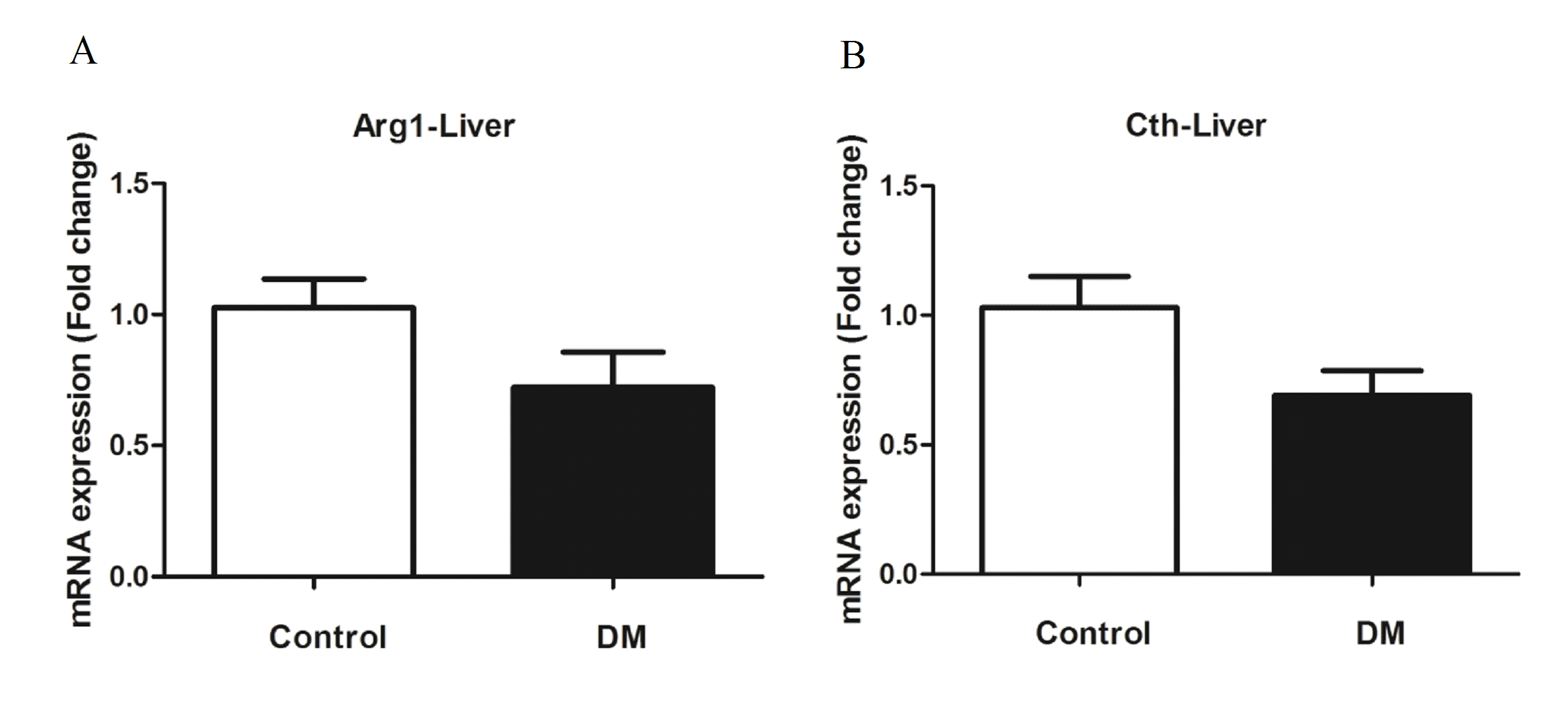

Supplement: S1 Fig — (TIF) [file pone.0191212.s001.tif]

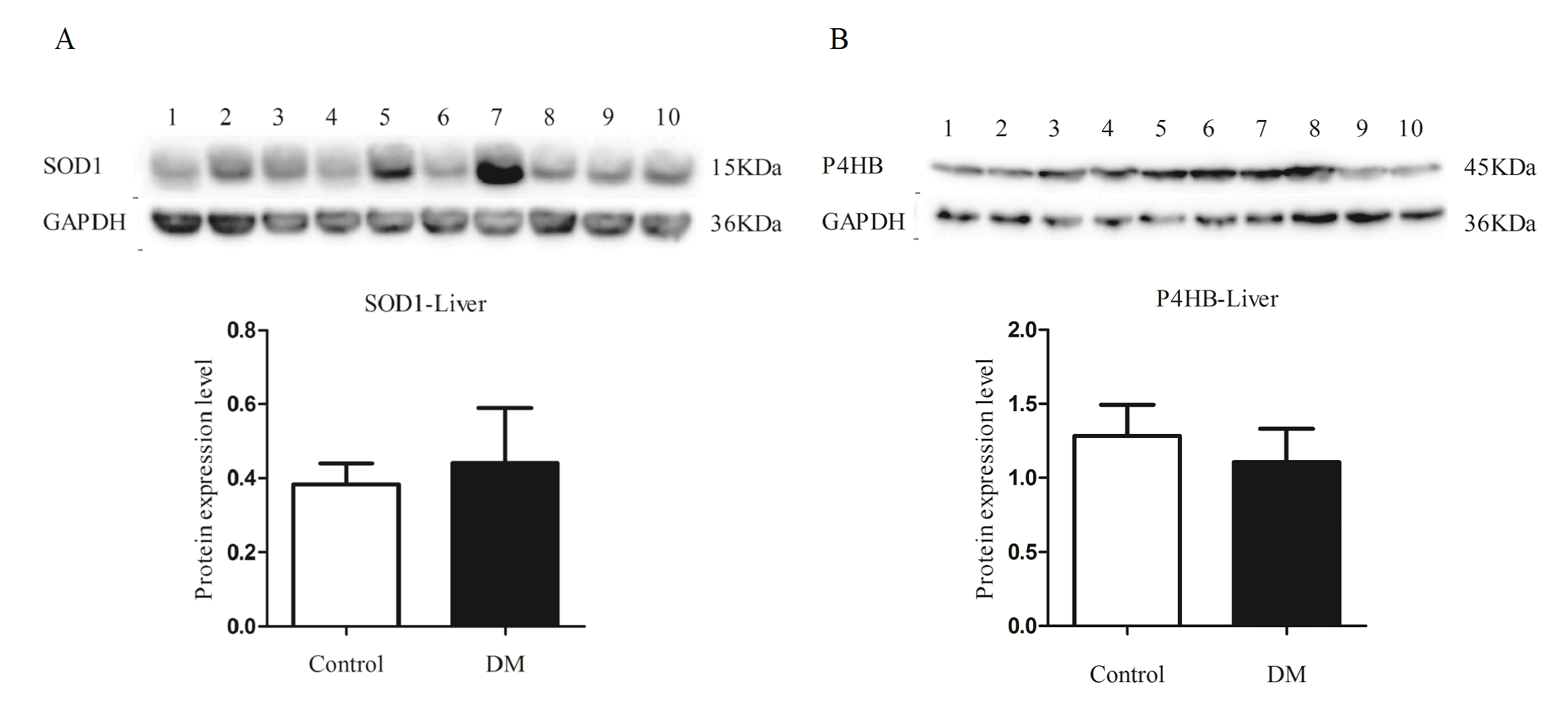

Supplement: S2 Fig — (TIF) [file pone.0191212.s002.tif]
