# Supplementary material for: Differential expression of genes identified by suppression subtractive hybridization in liver and adipose tissue of gerbils with diabetes
Source: PLoS One. 2018 Feb 2;13(2):e0191212. doi: 10.1371/journal.pone.0191212 (PMC5796689; doi:10.1371/journal.pone.0191212)
Supplement: S1 Table — (DOCX) [file pone.0191212.s003.docx]

**Supplementary Table 1.**User Id, dbEST Id, and GenBank Accn of ESTs from SSH libraries

| User ID | dbEST ID | GenBankAccn |
| --- | --- | --- |
| 1 | 79863188 | JZ923613 |
| 2 | 79863189 | JZ923614 |
| 3 | 79863190 | JZ923615 |
| 4 | 79863191 | JZ923616 |
| 5 | 79863192 | JZ923617 |
| 6 | 79863193 | JZ923618 |
| 7 | 79863194 | JZ923619 |
| 8 | 79863195 | JZ923620 |
| 9 | 79863196 | JZ923621 |
| 10 | 79863197 | JZ923622 |
| 11 | 79863198 | JZ923623 |
| 12 | 79863199 | JZ923624 |
| 13 | 79863200 | JZ923625 |
| 14 | 79863201 | JZ923626 |
| 15 | 79863202 | JZ923627 |
| 16 | 79863203 | JZ923628 |
| 17 | 79863204 | JZ923629 |
| 18 | 79863205 | JZ923630 |
| 19 | 79863206 | JZ923631 |
| 20 | 79863207 | JZ923632 |
| 21 | 79863208 | JZ923633 |
| 22 | 79863209 | JZ923634 |
| 23 | 79863210 | JZ923635 |
| 24 | 79863211 | JZ923636 |
| 25 | 79863212 | JZ923637 |
| 26 | 79863213 | JZ923638 |
| 27 | 79863214 | JZ923639 |
| 28 | 79863215 | JZ923640 |
| 29 | 79863216 | JZ923641 |
| 30 | 79863217 | JZ923642 |
| 31 | 79863218 | JZ923643 |
| 32 | 79863219 | JZ923644 |
| 33 | 79863247 | JZ923672 |
| 34 | 79863220 | JZ923645 |
| 35 | 79863221 | JZ923646 |
| 36 | 79863222 | JZ923647 |
| 37 | 79863223 | JZ923648 |
| 38 | 79863224 | JZ923649 |
| 39 | 79863248 | JZ923673 |
| 40 | 79863225 | JZ923650 |
| 41 | 79863249 | JZ923674 |
| 42 | 79863226 | JZ923651 |
| 43 | 79863227 | JZ923652 |
| 44 | 79863228 | JZ923653 |
| 45 | 79863229 | JZ923654 |
| 46 | 79863230 | JZ923655 |
| 47 | 79863231 | JZ923656 |
| 48 | 79863232 | JZ923657 |
| 49 | 79863233 | JZ923658 |
| 50 | 79863234 | JZ923659 |
| 51 | 79863235 | JZ923660 |
| 52 | 79863236 | JZ923661 |
| 53 | 79863237 | JZ923662 |
| 54 | 79863238 | JZ923663 |
| 55 | 79863239 | JZ923664 |
| 56 | 79863240 | JZ923665 |
| 57 | 79863241 | JZ923666 |
| 58 | 79863242 | JZ923667 |
| 59 | 79863243 | JZ923668 |
| 60 | 79863244 | JZ923669 |
| 61 | 79863245 | JZ923670 |
| 62 | 79863246 | JZ923671 |
