# Supplementary material for: Differential expression of genes identified by suppression subtractive hybridization in liver and adipose tissue of gerbils with diabetes
Source: PLoS One. 2018 Feb 2;13(2):e0191212. doi: 10.1371/journal.pone.0191212 (PMC5796689; doi:10.1371/journal.pone.0191212)
Supplement: S1 File — (ZIP) [file pone.0191212.s004.zip › Un-altered western blot images-Figure 2.pptx]

## Slide 1
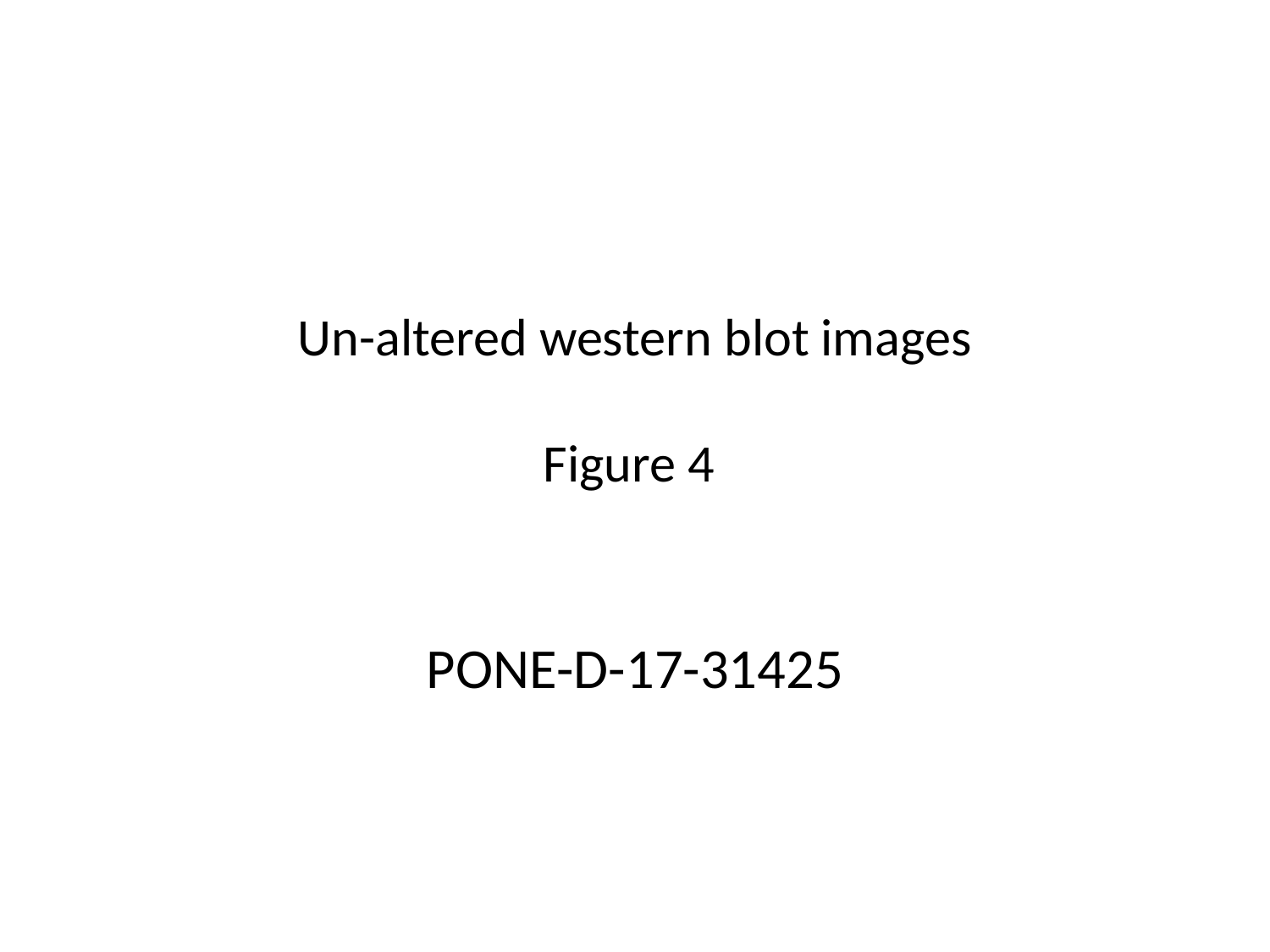

# Un-altered western blot images Figure 4
PONE-D-17-31425

## Slide 2
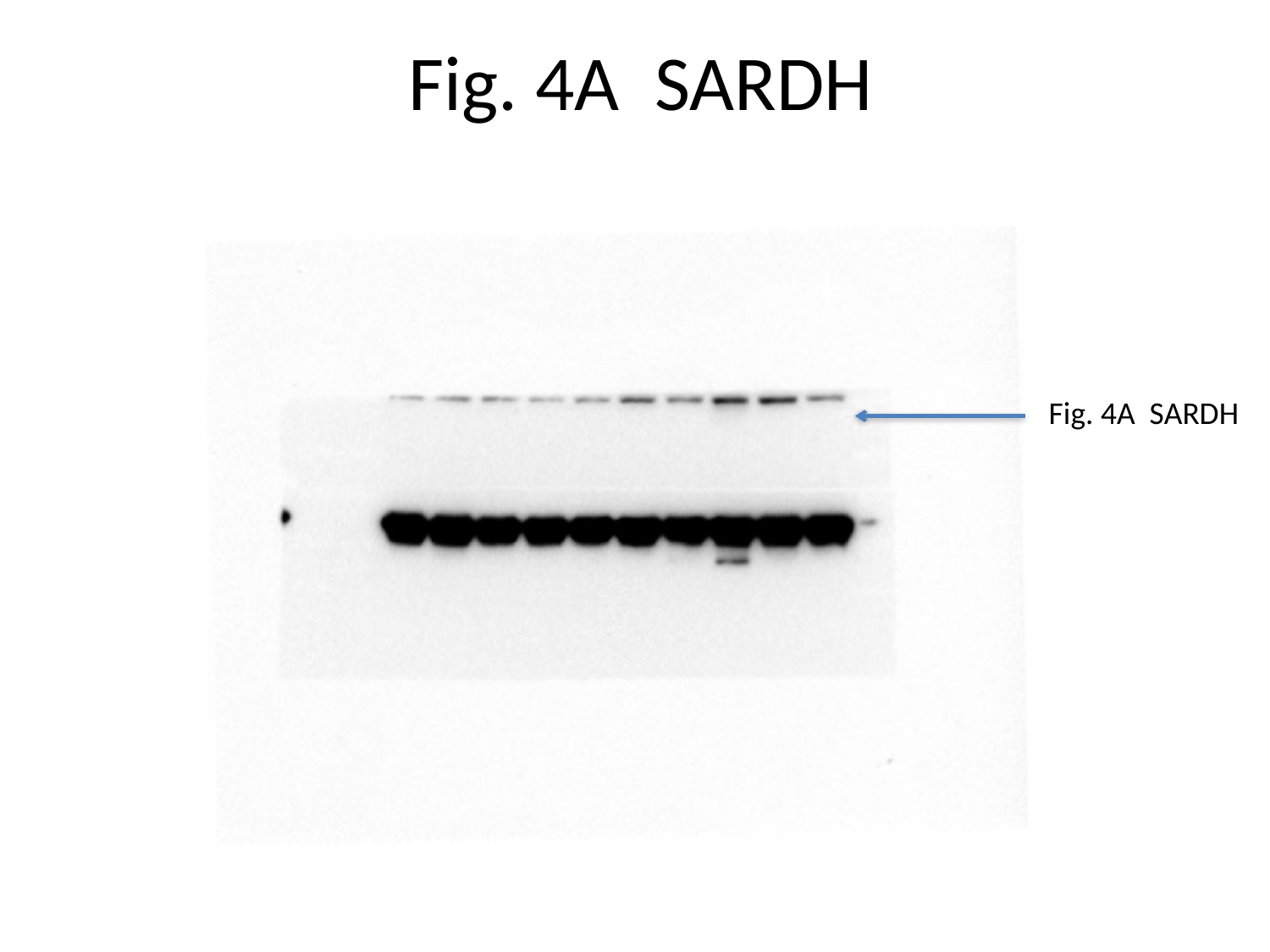

# Fig. 4A SARDH
Fig. 4A SARDH

## Slide 3
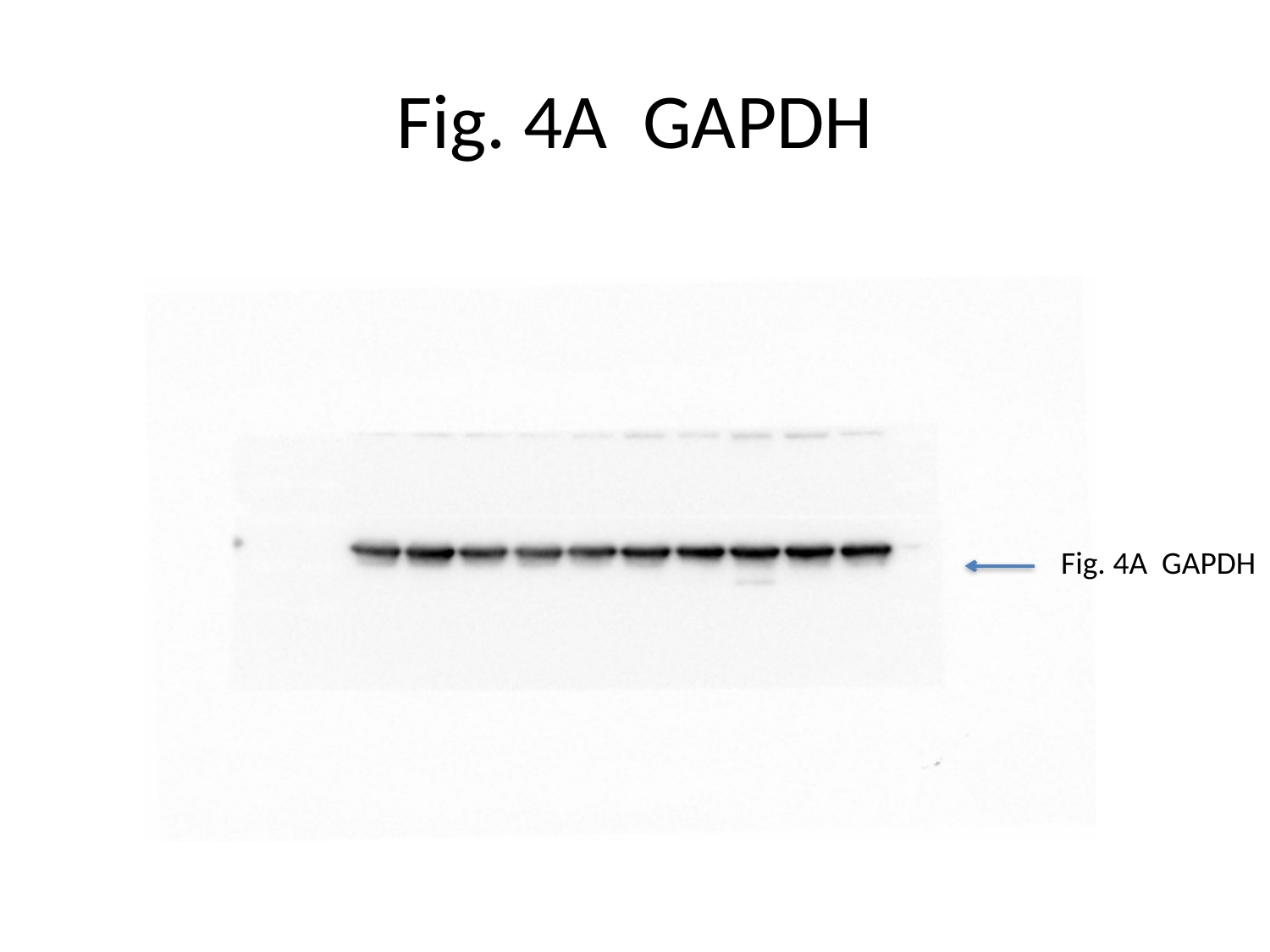

# Fig. 4A GAPDH
Fig. 4A GAPDH

## Slide 4
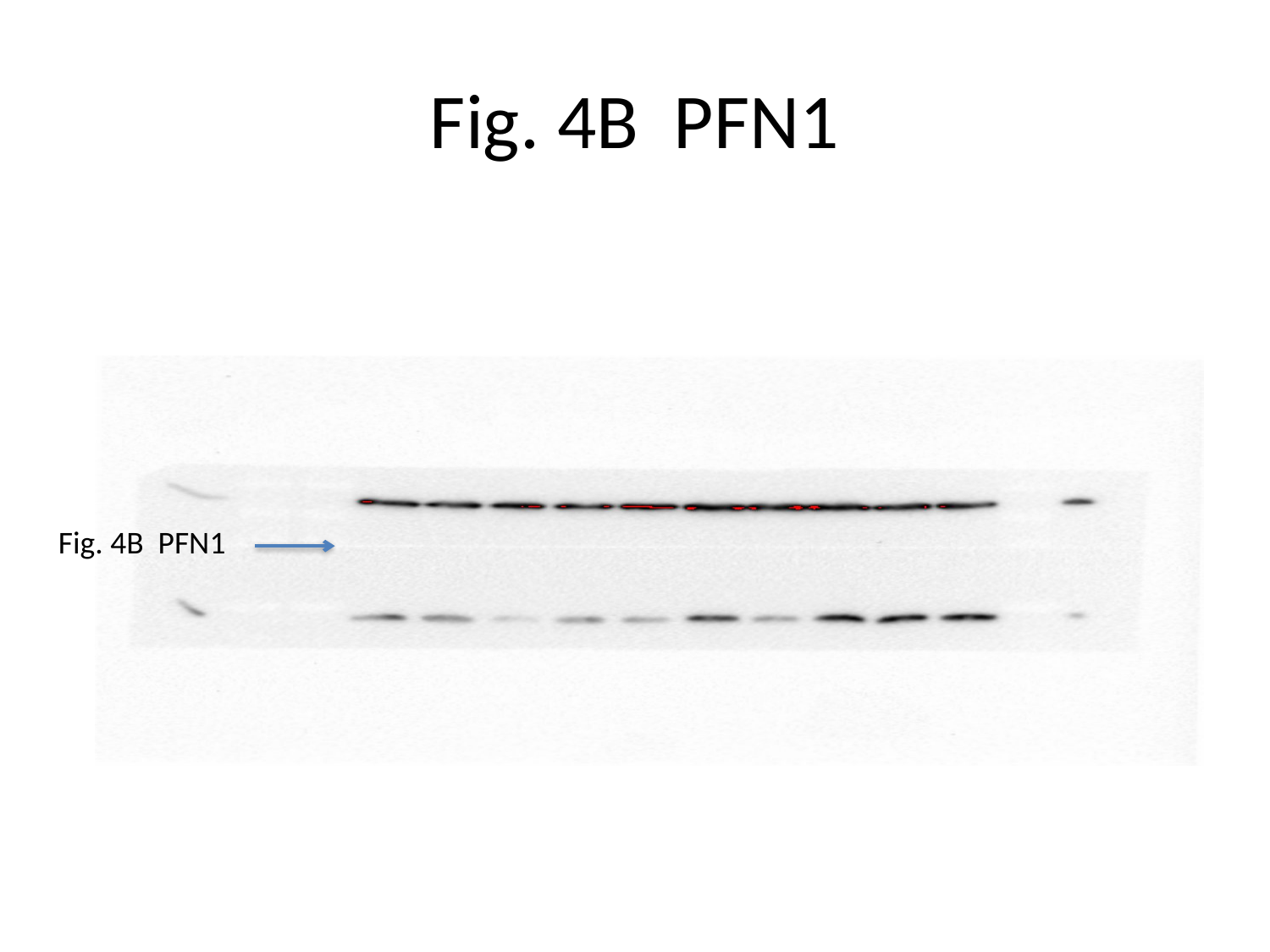

# Fig. 4B PFN1
Fig. 4B PFN1

## Slide 5
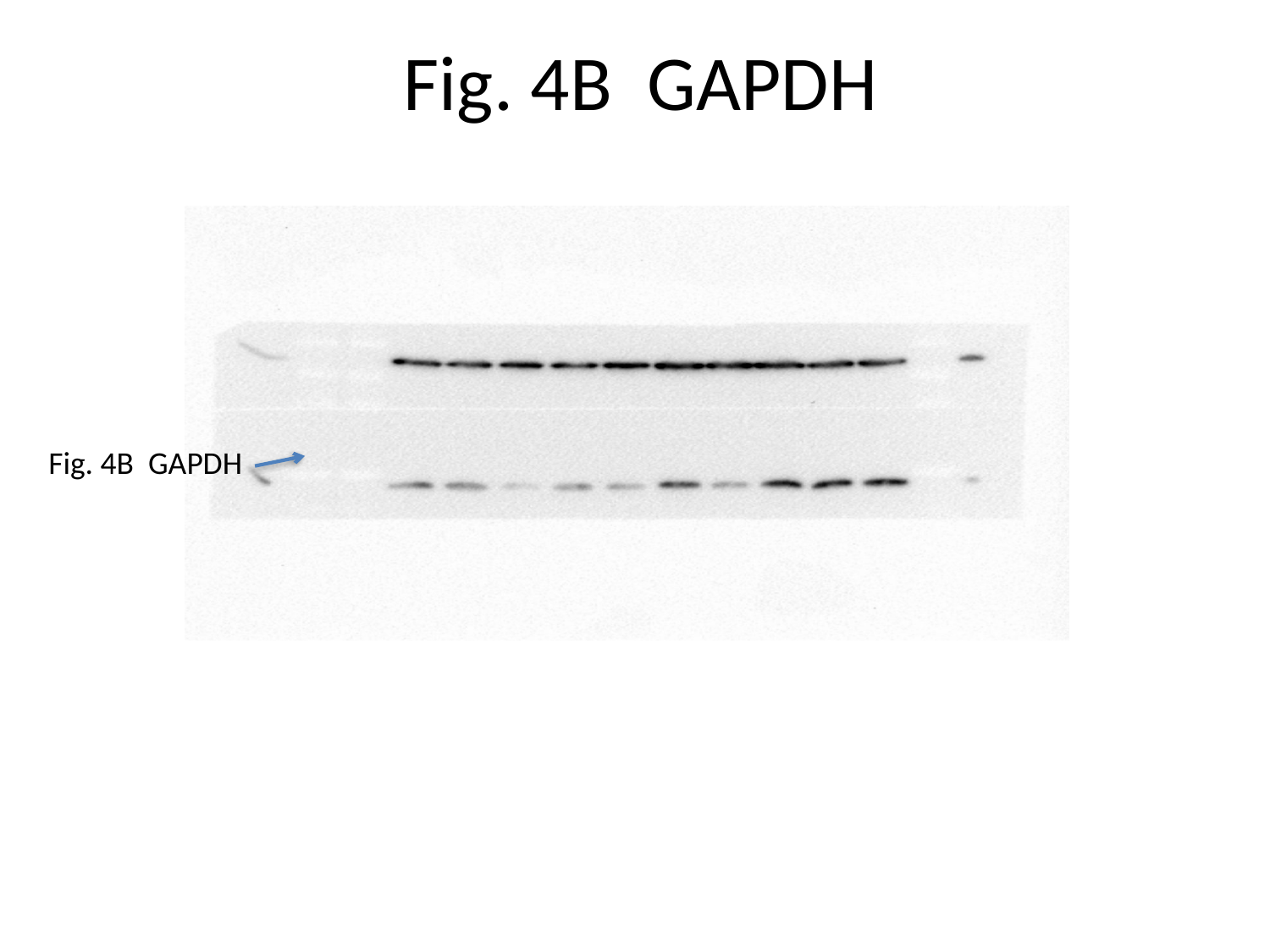

# Fig. 4B GAPDH
Fig. 4B GAPDH

## Slide 6
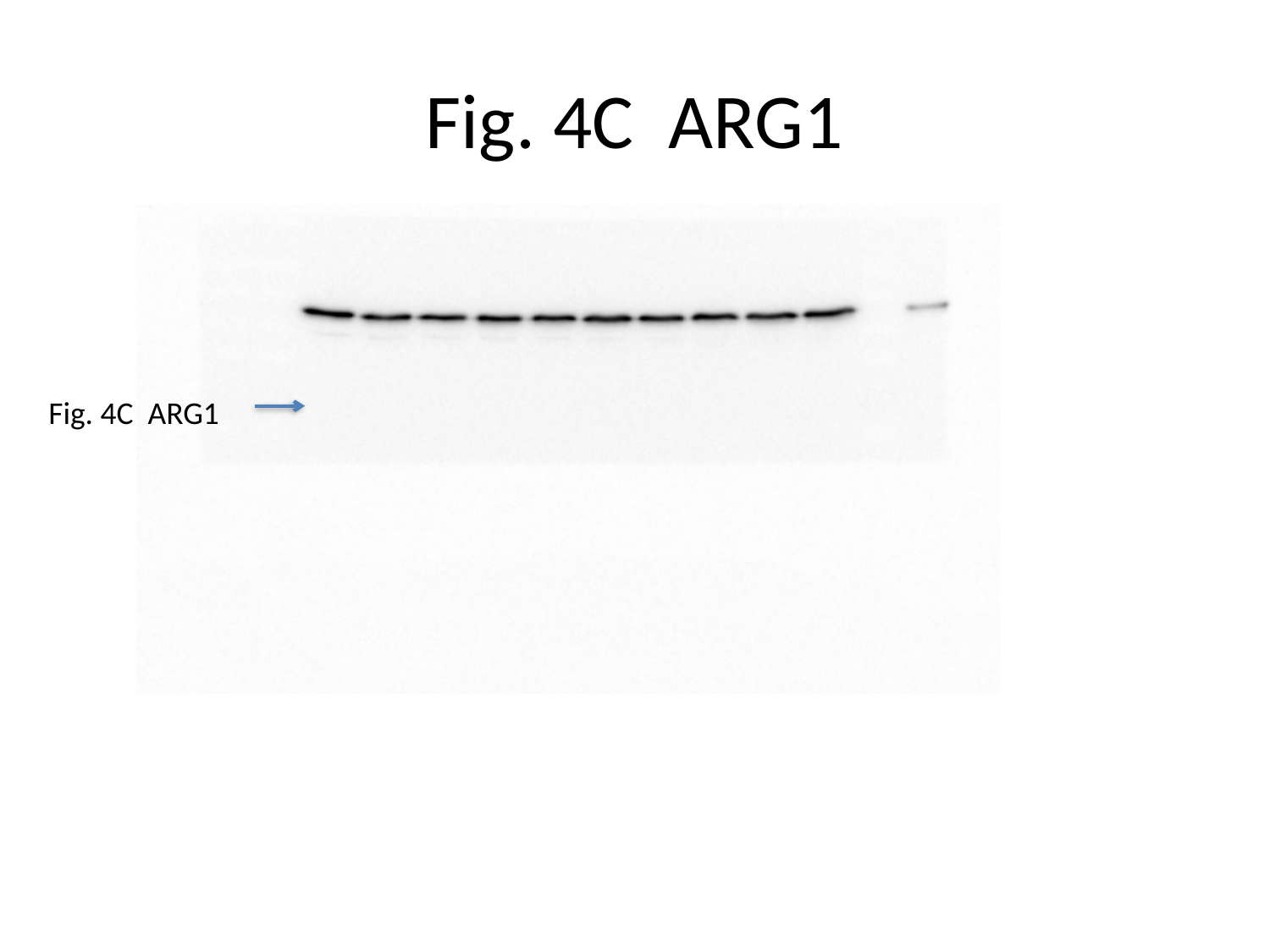

# Fig. 4C ARG1
Fig. 4C ARG1

## Slide 7
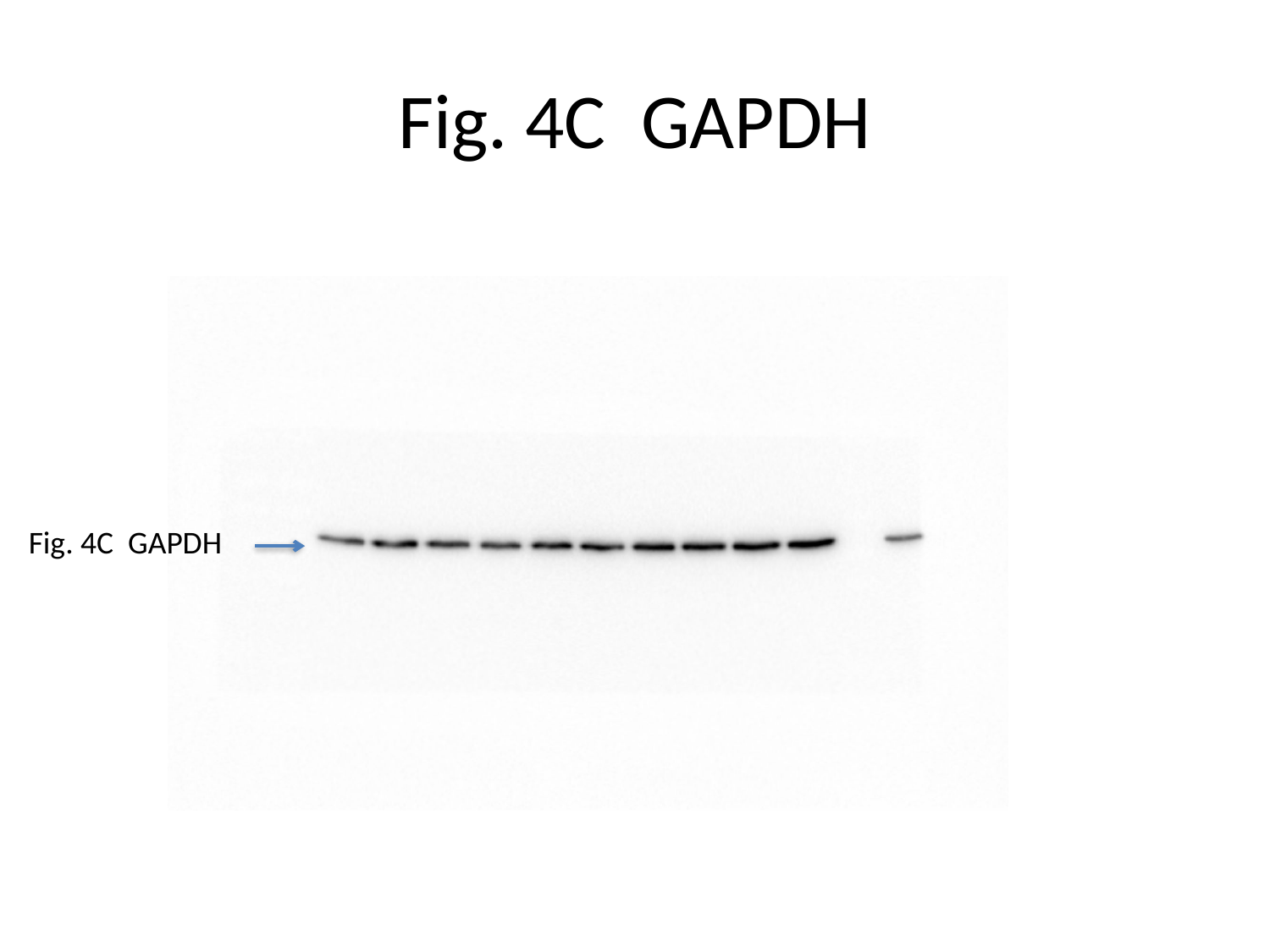

# Fig. 4C GAPDH
Fig. 4C GAPDH

## Slide 8
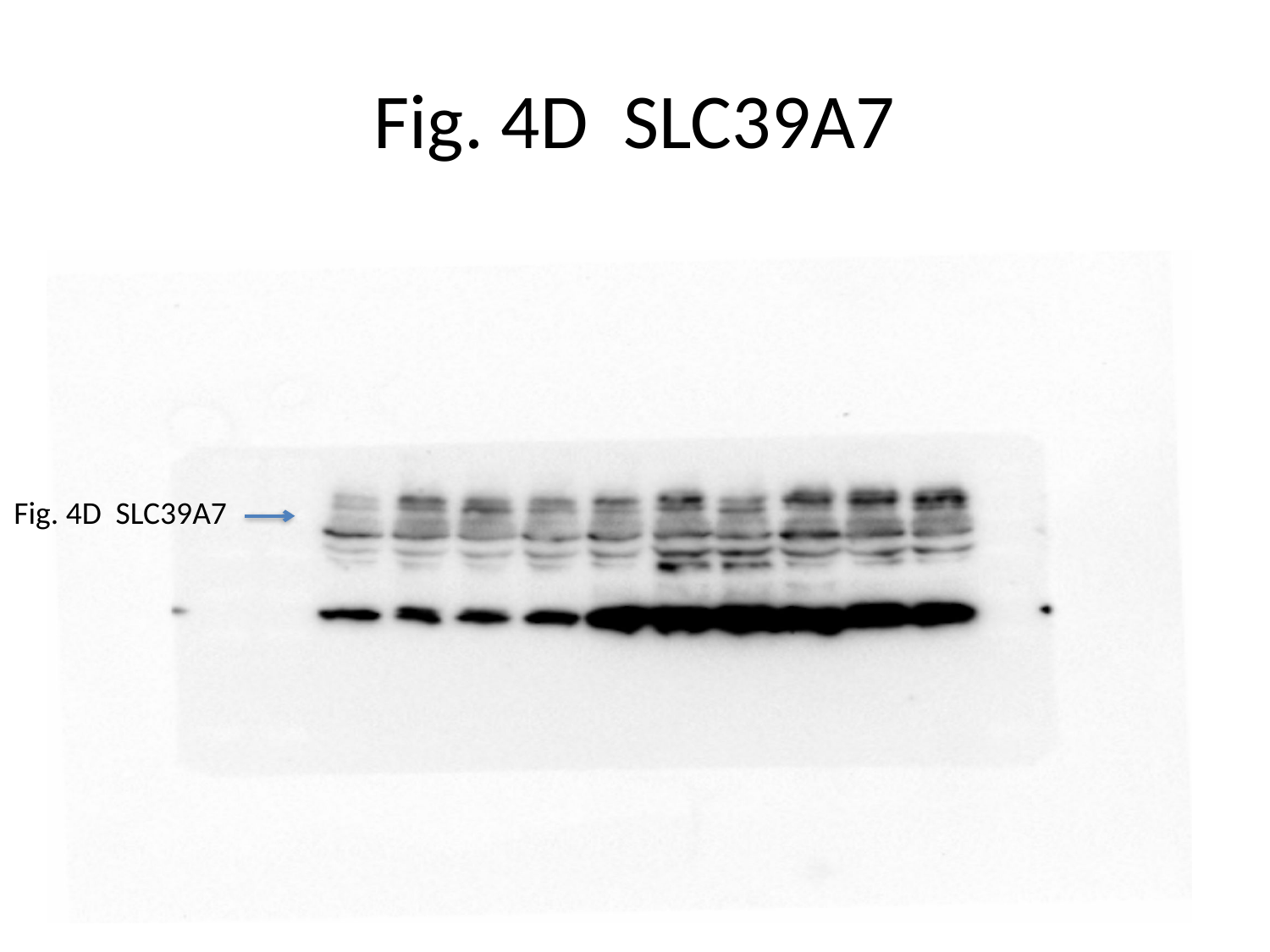

# Fig. 4D SLC39A7
Fig. 4D SLC39A7

## Slide 9
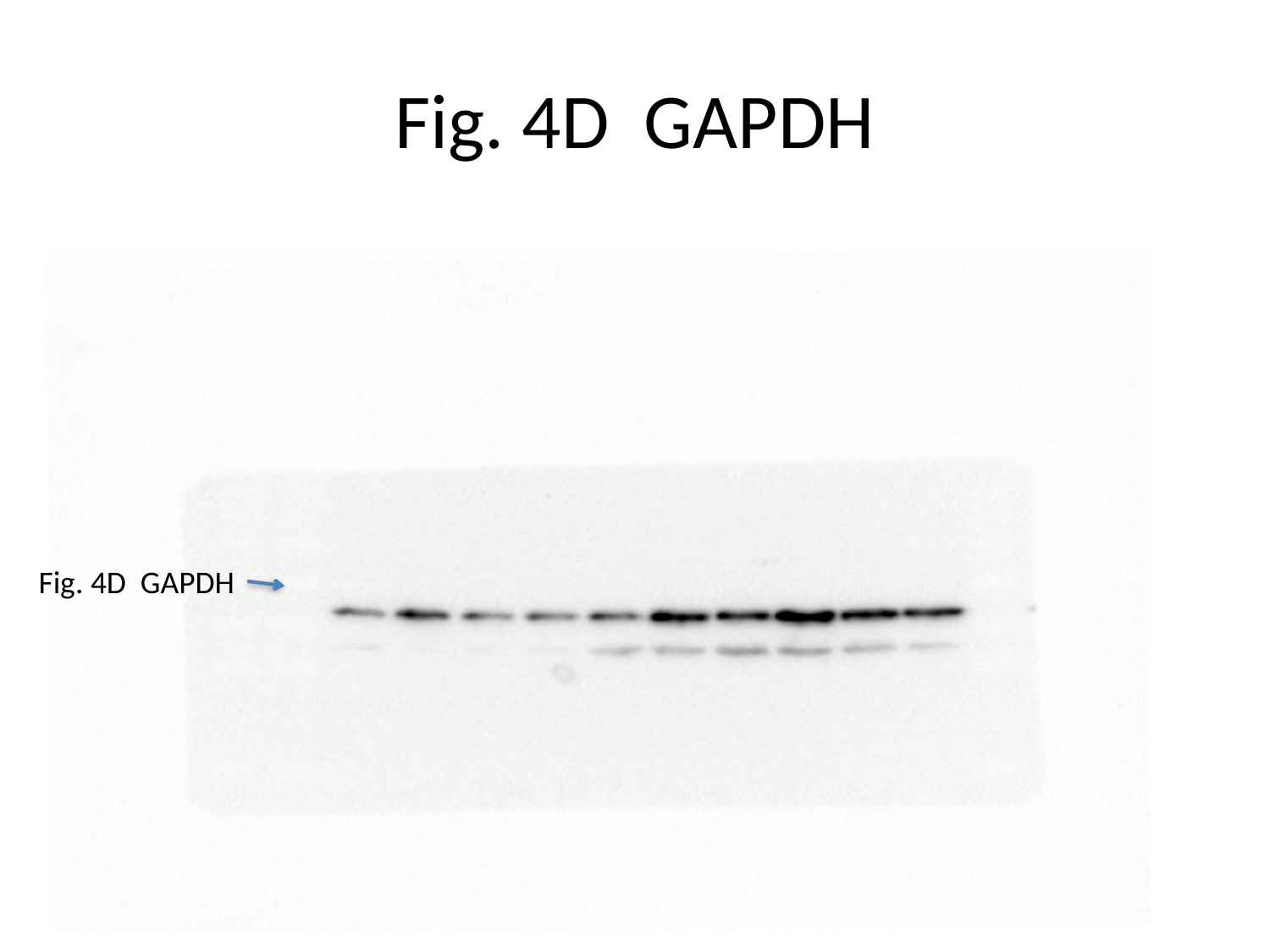

# Fig. 4D GAPDH
Fig. 4D GAPDH

## Slide 10
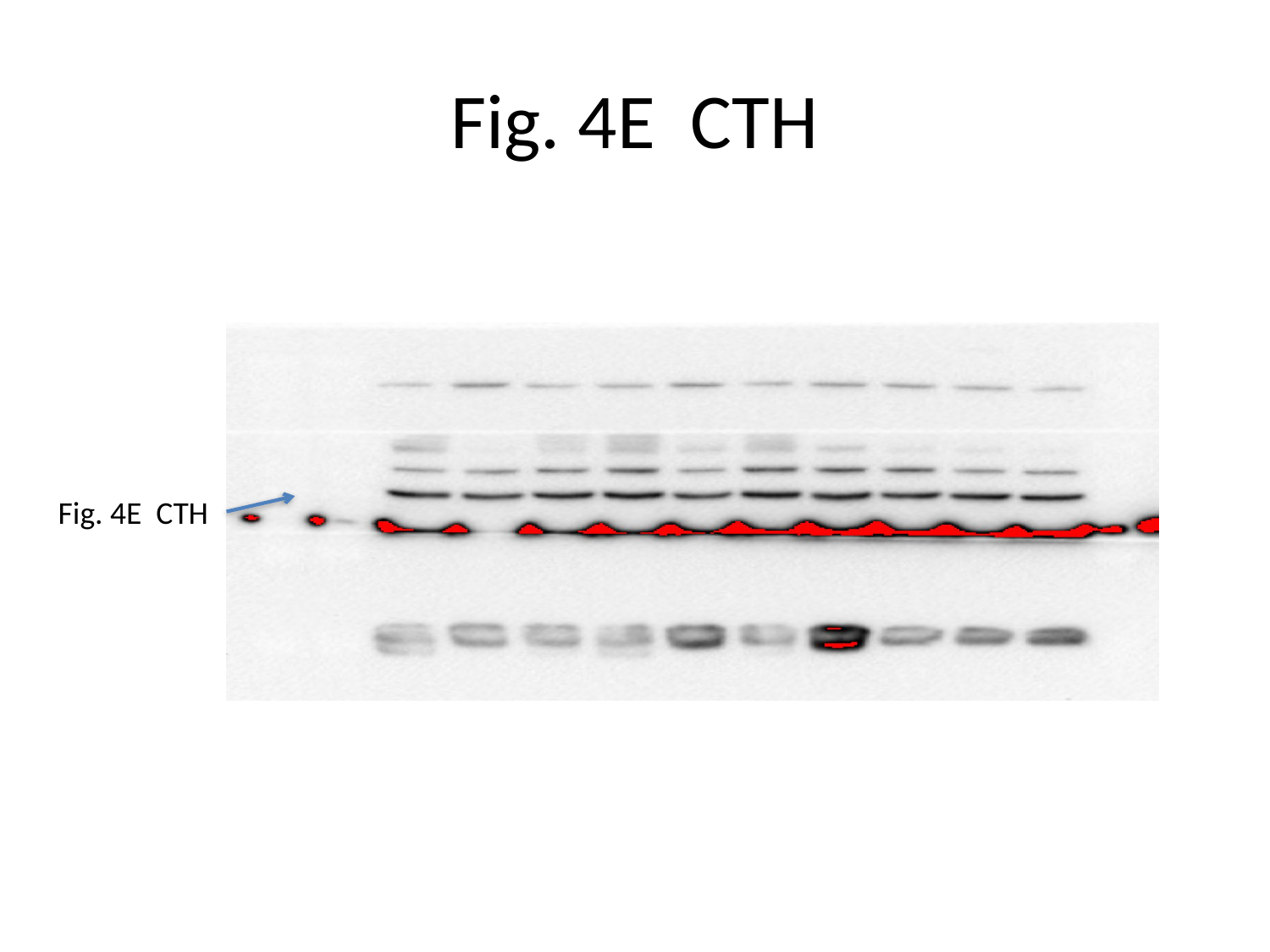

# Fig. 4E CTH
Fig. 4E CTH

## Slide 11
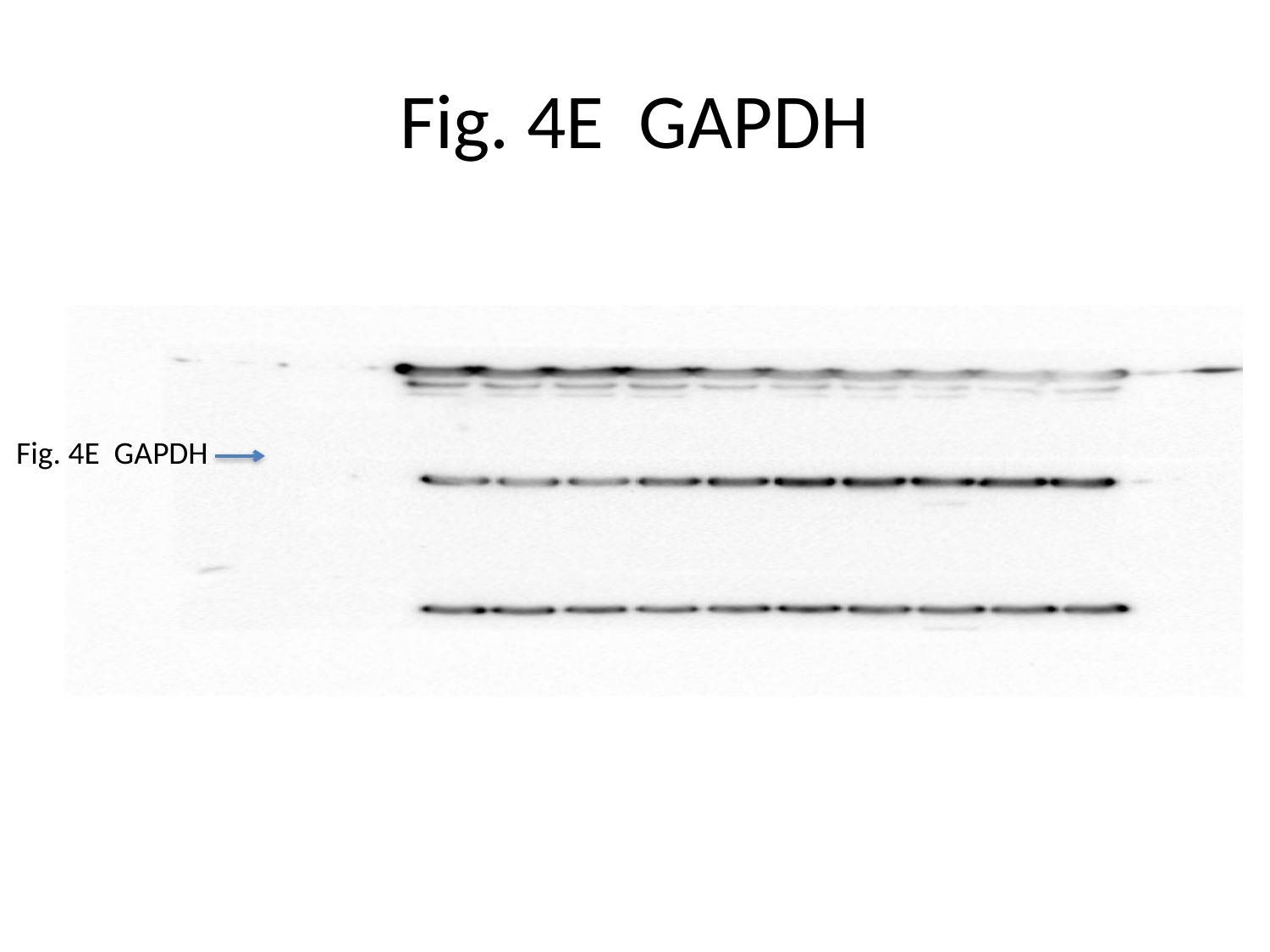

# Fig. 4E GAPDH
Fig. 4E GAPDH

## Slide 12
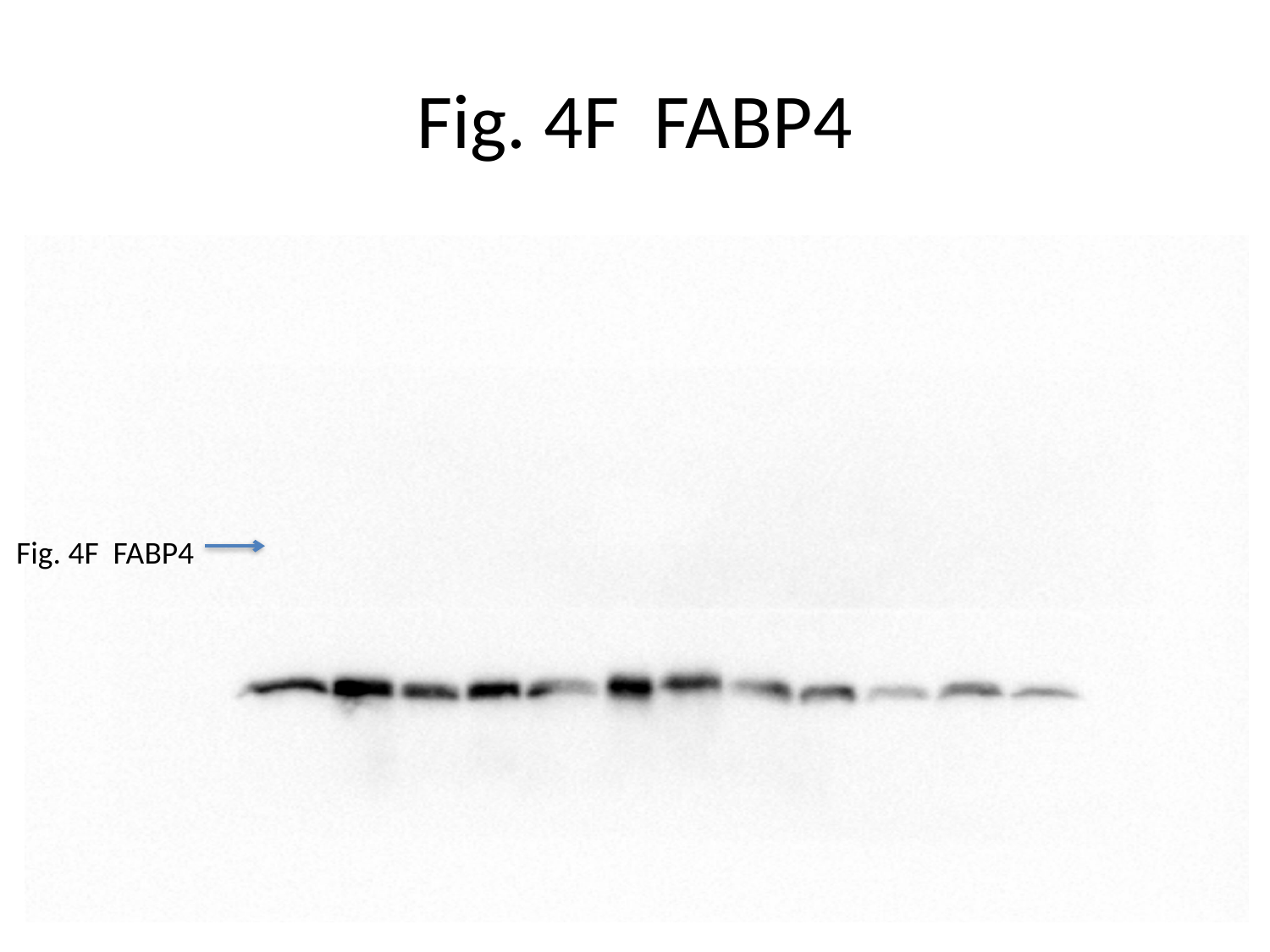

# Fig. 4F FABP4
Fig. 4F FABP4

## Slide 13
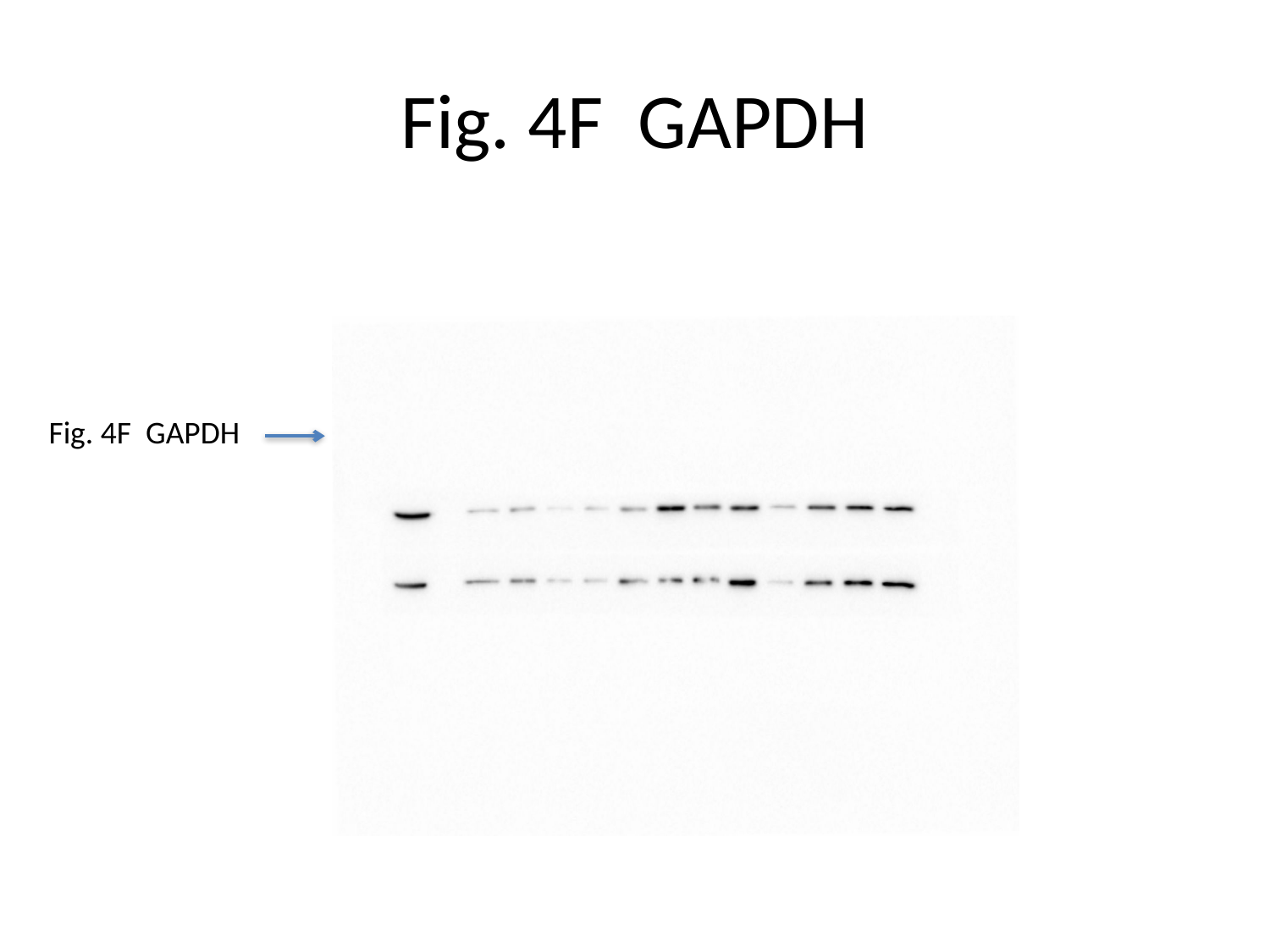

# Fig. 4F GAPDH
Fig. 4F GAPDH
