# Supplementary material for: Differential expression of genes identified by suppression subtractive hybridization in liver and adipose tissue of gerbils with diabetes
Source: PLoS One. 2018 Feb 2;13(2):e0191212. doi: 10.1371/journal.pone.0191212 (PMC5796689; doi:10.1371/journal.pone.0191212)
Supplement: S2 File — (ZIP) [file pone.0191212.s005.zip › Un-altered western blot images-Supplementary Figure 2.pptx]

## Slide 1
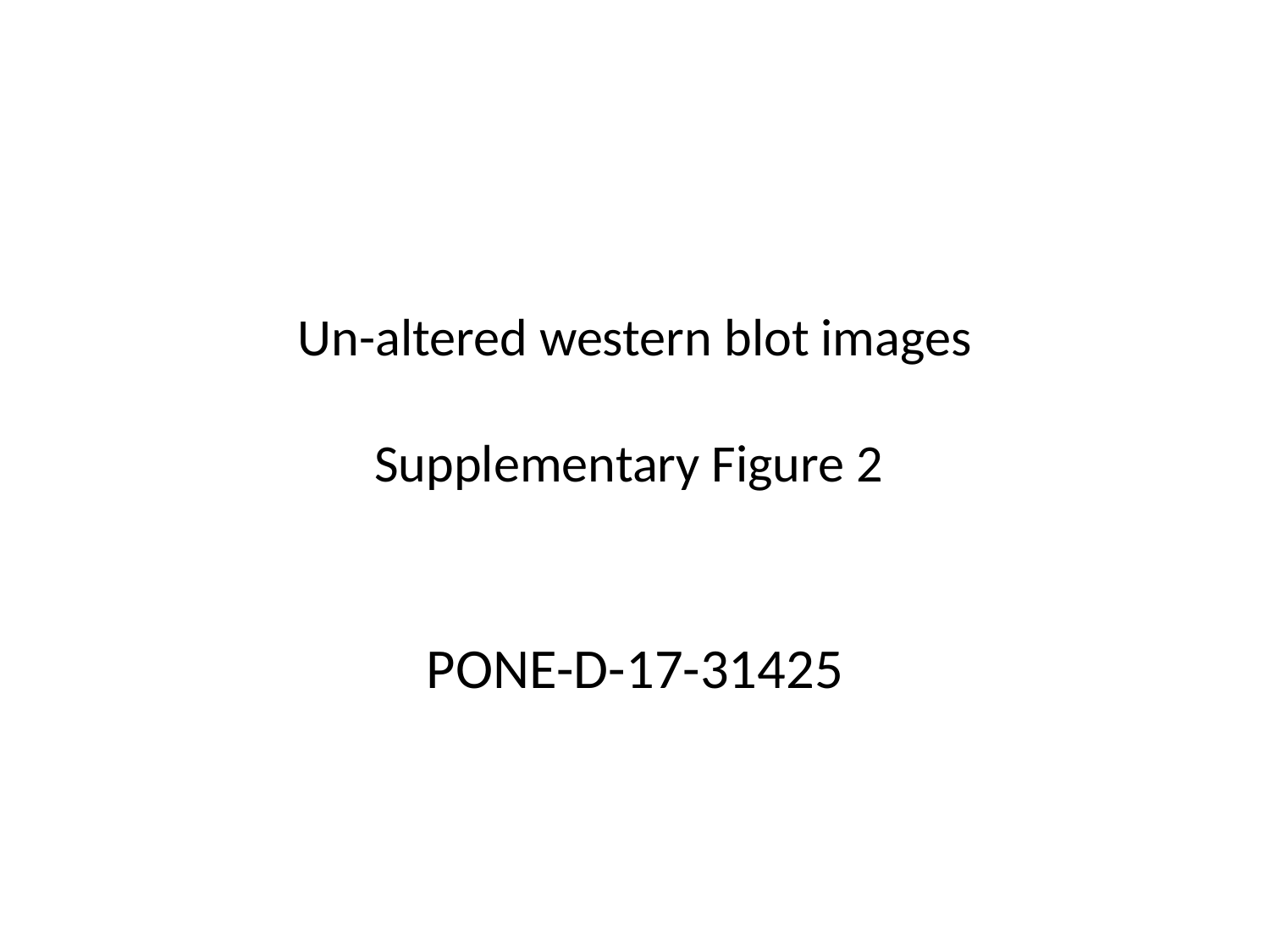

# Un-altered western blot images Supplementary Figure 2
PONE-D-17-31425

## Slide 2
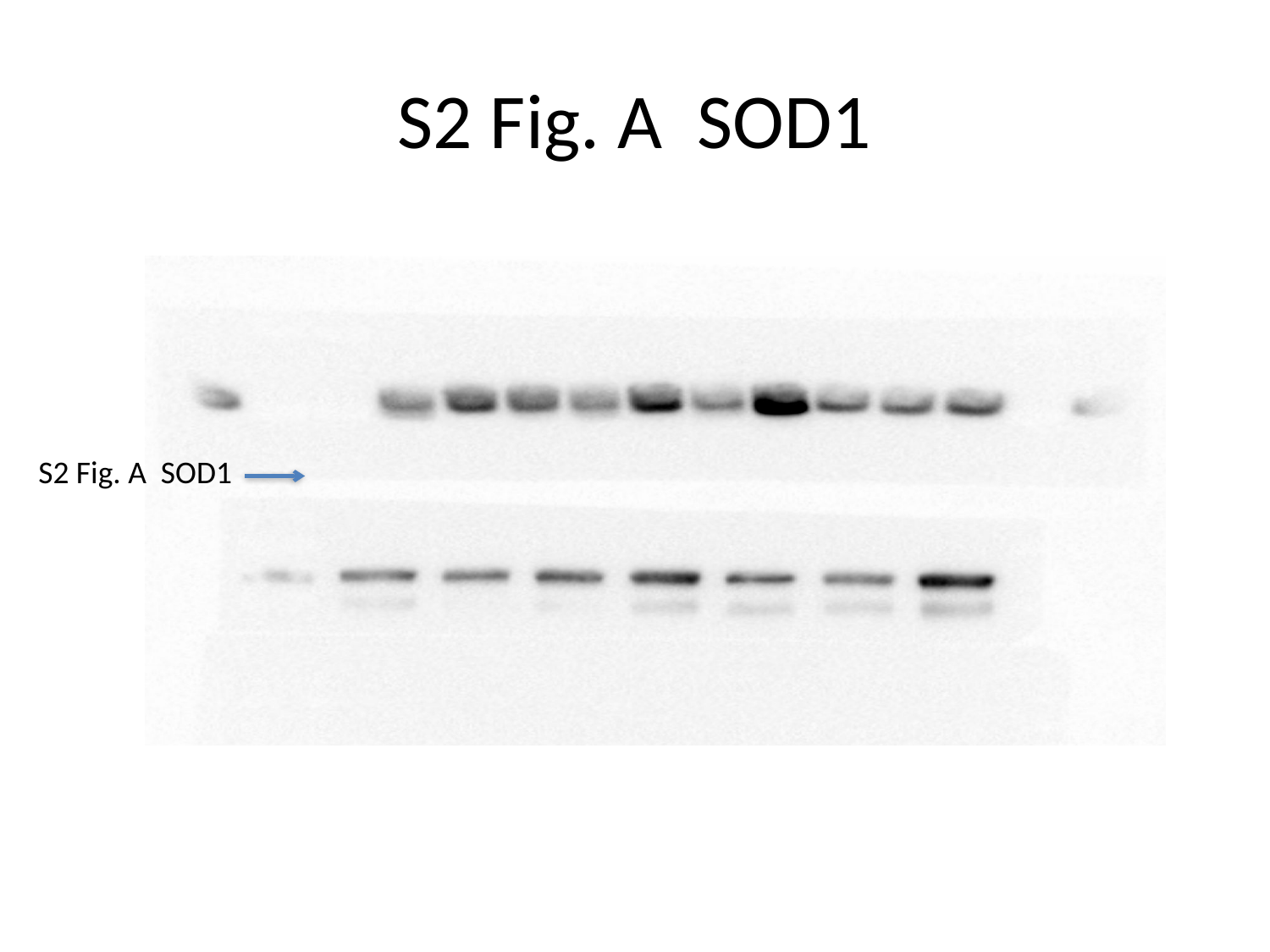

# S2 Fig. A SOD1
S2 Fig. A SOD1

## Slide 3
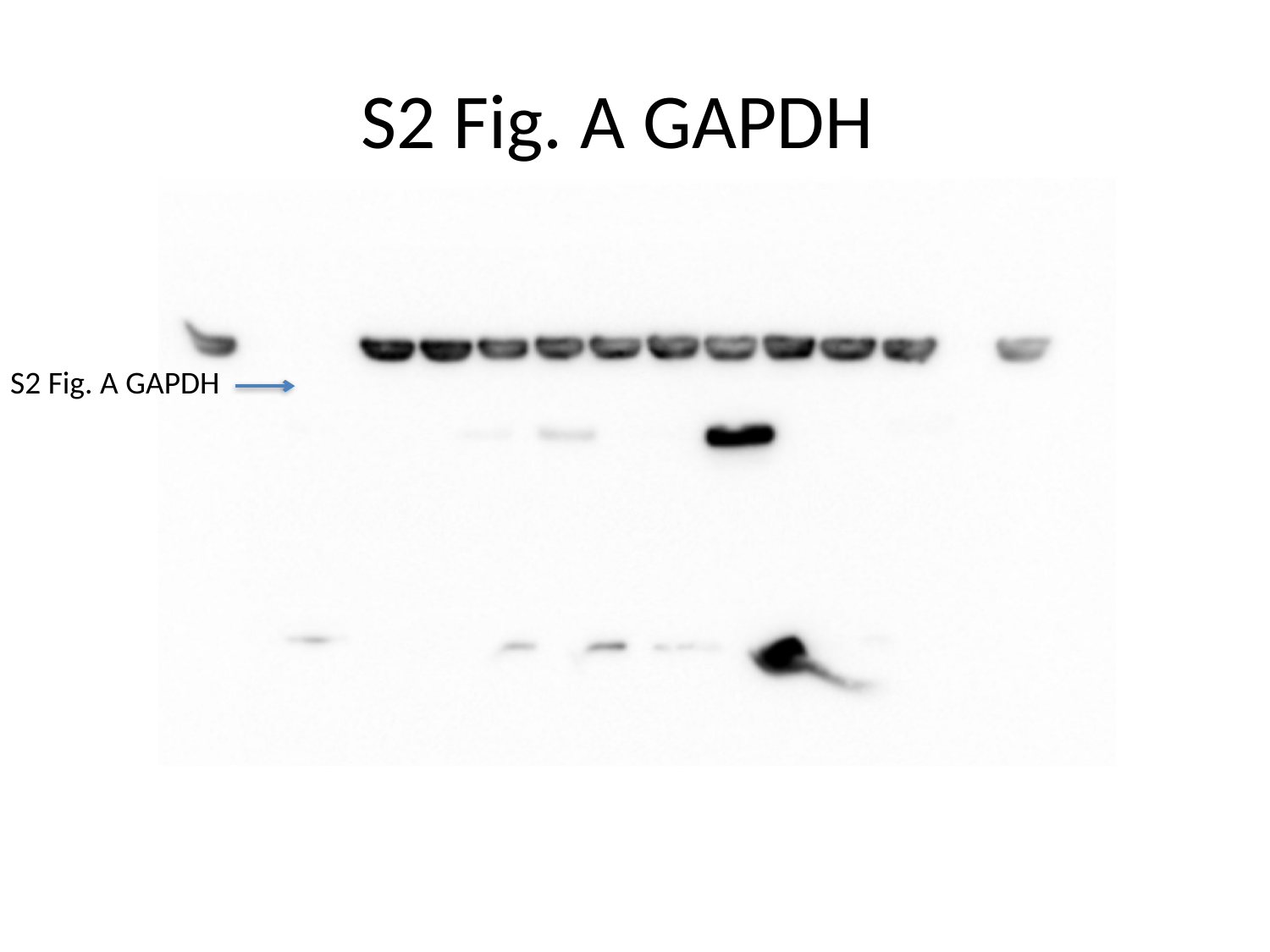

# S2 Fig. A GAPDH
S2 Fig. A GAPDH

## Slide 4
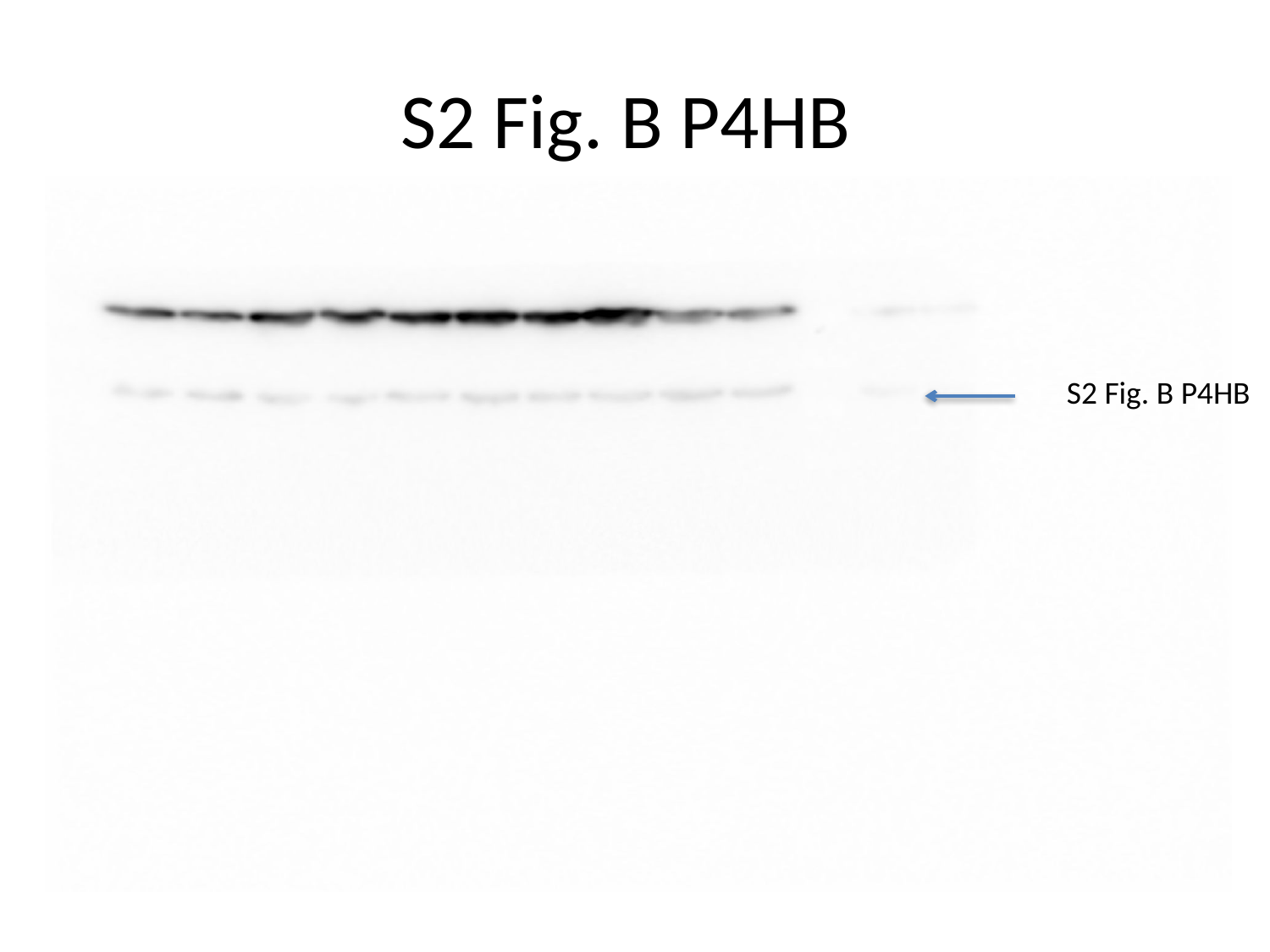

# S2 Fig. B P4HB
S2 Fig. B P4HB

## Slide 5
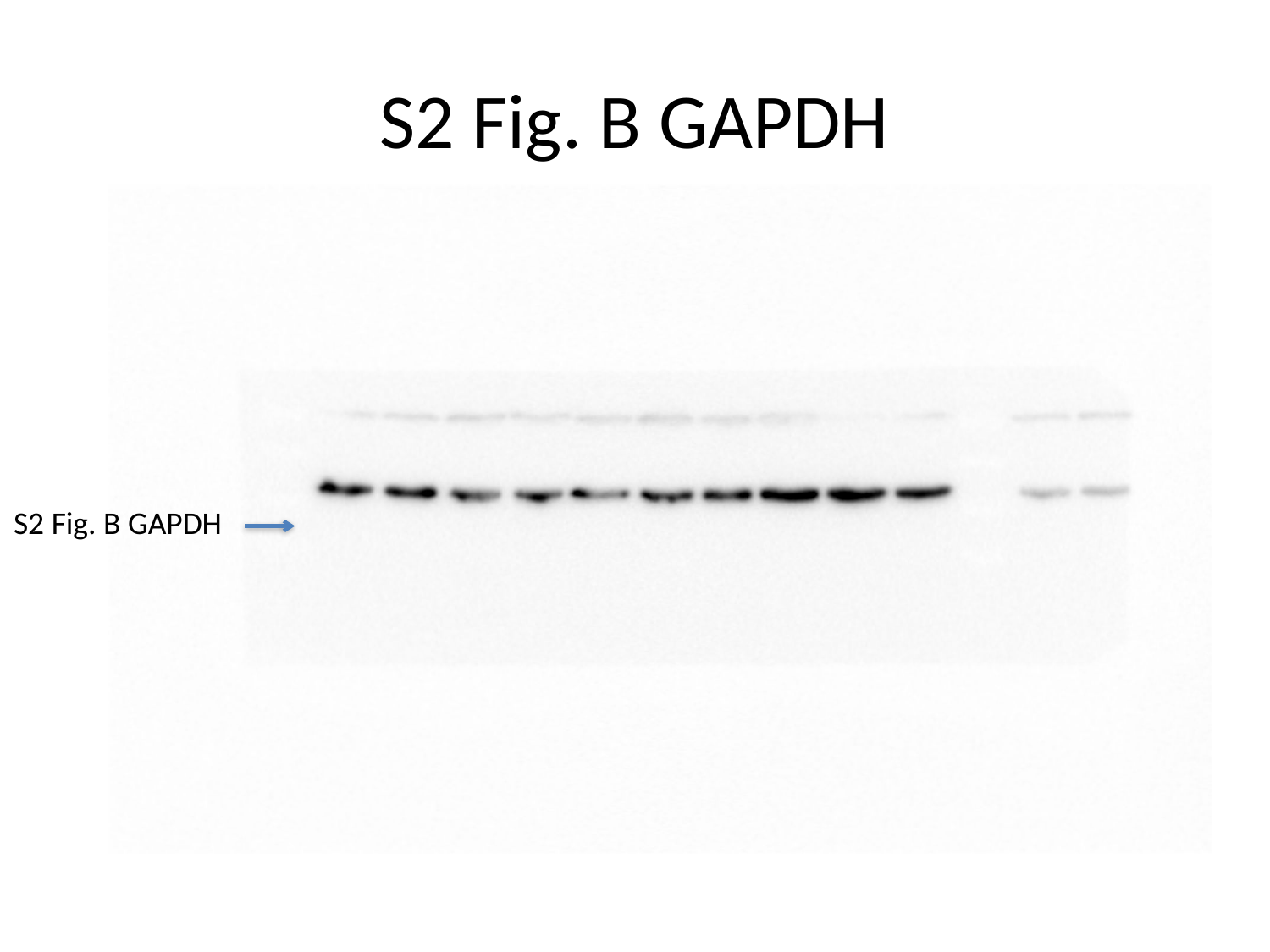

# S2 Fig. B GAPDH
S2 Fig. B GAPDH
